# Dynamic transcriptome landscape of Asian domestic honeybee (*Apis cerana*) embryonic development revealed by high-quality RNA sequencing

**DOI:** 10.1186/s12861-018-0169-1

**Published:** 2018-04-13

**Authors:** Xiaofen Hu, Li Ke, Zilong Wang, Zhijiang Zeng

**Affiliations:** Honeybee Research Institute, Jiangxi Agricultural University, Nanchang, 330045 Jiangxi China

**Keywords:** *Apis cerana*, Embryonic transcriptome, Dynamic regulation of transcripts, Embryonic development

## Abstract

**Background:**

Honeybee development consists of four stages: embryo, larva, pupa and adult. Embryogenesis, a key process of cell division and differentiation, takes 3 days in honeybees. However, the embryonic transcriptome and the dynamic regulation of embryonic transcription are still largely uncharacterized in honeybees, especially in the Asian honeybee (*Apis cerana*). Here, we employed high-quality RNA-seq to explore the transcriptome of Asian honeybee embryos at three ages, approximately 24, 48 and 72 h (referred to as Day1, Day2 and Day3, respectively).

**Results:**

Nine embryo samples, three from each age, were collected for RNA-seq. According to the staging scheme of honeybee embryos and the morphological features we observed, our Day1, Day2 and Day3 embryos likely corresponded to the late stage four, stage eight and stage ten development stages, respectively. Hierarchical clustering and principal component analysis showed that same-age samples were grouped together, and the Day2 samples had a closer relationship with the Day3 samples than the Day1 samples. Finally, a total of 18,284 genes harboring 55,646 transcripts were detected in the *A. cerana* embryos, of which 44.5% consisted of the core transcriptome shared by all three ages of embryos. A total of 4088 upregulated and 3046 downregulated genes were identified among the three embryo ages, of which 2010, 3177 and 1528 genes were upregulated and 2088, 2294 and 303 genes were downregulated from Day1 to Day2, from Day1 to Day3 and from Day2 to Day3, respectively. The downregulated genes were mostly involved in cellular, biosynthetic and metabolic processes, gene expression and protein localization, and macromolecule modification; the upregulated genes mainly participated in cell development and differentiation, tissue, organ and system development, and morphogenesis. Interestingly, several biological processes related to the response to and detection of light stimuli were enriched in the first-day *A. cerana* embryogenesis but not in the *Apis mellifera* embryogenesis, which was valuable for further investigations.

**Conclusions:**

Our transcriptomic data substantially expand the number of known transcribed elements in the *A. cerana* genome and provide a high-quality view of the transcriptome dynamics of *A. cerana* embryonic development.

**Electronic supplementary material:**

The online version of this article (10.1186/s12861-018-0169-1) contains supplementary material, which is available to authorized users.

## Background

Bees are flying insects that play important roles in pollination and are keystone species supporting critical ecosystem services [1]; honeybees represent a small fraction of the known bee species [2]. The best-known honeybees are the European honeybee (*Apis mellifera*) and Asian honeybee (*Apis cerana*). Both European and Asian honeybees are eusocial flying insects that have a great impact on the global ecological environment and can be domesticated for honey production and crop pollination [3, 4]. Additionally, the honeybee is an excellent model animal to elucidate the molecular and neural mechanisms underlying insect social behaviors [5, 6] and serves as a model insect for developmental genetics [7].

Compared to Asian honeybees, European honeybees are more widely used around the globe and can produce more honey products. However, Asian honeybees have several advantages over European honeybees: (1) Asian honeybees have a good resistance to low temperatures that can be lethal to European honeybees [8]; (2) Asian honeybees are better able to collect honey in sporadic nectar sources and have a longer honey collection period [9]; (3) Asian honeybees are more active and more suitable to raise in mountain areas; and (4) Asian honeybees possess a strong resilience to the ectoparasitic mite [10], which has a catastrophic effect on the population of European honeybees [11].

The honeybee development consists of four life cycle phases: embryo, larva, pupa and adult [3, 12]. Among these phases, embryogenesis, which is precisely controlled and modulated by both environmental and intracellular signals, is the first and the most important stage. During the embryogenesis period, the rudimentary organs of the adult honeybees are gradually formed [12, 13], and gene expression in the embryonic stage contributes greatly to the adult morphology, appearance and behavior [14–16].

Since the latter half of the nineteenth century, morphological studies on honeybee embryogenesis have been carried out [7], providing plentiful morphological data and detailing the staging scheme of honeybee embryos [13, 17, 18]. In the late 1980s, molecular studies investigating honeybee embryonic development were conducted [7], providing a solid foundation to further study honeybee embryogenesis [19–25]. Currently, visualization tools and the genetic manipulation of honeybee embryos have been developed, including in situ hybridization [26], immunohistochemistry [27], RNA interference [28, 29], transgenesis with the transposon piggyBac [30], and genome editing by the CRISPR/Cas9 method [31]. Short-term [32], long-term [33] and immortalized cell lines [34] have been successfully developed, and non-*Apis* genes can be expressed in cultured embryonic cells [35]. Meanwhile, high-throughput sequencing, also known as next-generation sequencing (NGS), has become essential for modern biological research, and it has been widely applied to honeybee genome [36, 37], transcriptome [38, 39] and metagenome [40] analyses. Moreover, mass spectrometry (MS)-based proteomics has also been developed as a powerful technology providing a wider view of honeybee proteomes and their changes [41, 42]. Altogether, these current technologies have elevated the study of honeybee embryogenesis to a new level.

The genome of *A. mellifera* was published in 2006 [36]. More recently, the genome and gene annotation of *A. mellifera* were upgraded and are now nearly complete [43]. Several transcriptomic and proteomic studies of *A. mellifera* have been performed to elucidate honeybee embryogenesis [38, 41, 42, 44]. However, the first draft genome of *A. cerana* was not assembled until 2015 [37]; the molecular mechanism of *A. cerana* embryogenesis is still poorly understood and needs further exploration.

Recently, transcriptome analyses were employed to investigate the different gene expression trends between workers and Queens in *A. cerana* [39]. Transcriptome analyses of haploid and diploid embryos have also been applied to reveal early zygotic transcription during cleavage in *A. mellifera* [38]. Deep sequencing and expression of microRNAs from early *A. mellifera* embryos were used to reveal the role of microRNAs in regulating early embryonic patterning [44]. However, the gene expression strategies during the embryonic development of Asian honeybees are still largely unknown. Here, we collected nine samples at the embryonic ages of approximately 24 (Day1), 48 (Day2), and 72 h (Day3) and employed high-coverage RNA-seq to explore their transcriptomes. Our study provided a high-quality view of the transcriptome dynamics of Asian honeybee embryogenesis and is an important complement to the embryonic development of other social insect species.

## Methods

### Ethics statement

All animal procedures were performed in accordance with the guidelines developed by the China Council on Animal Care, and protocols were approved by the Animal Care and Use Committee of Jiangxi Agricultural University, China.

### Sample collection

The embryo samples of *A. cerana* were obtained from three honeybee colonies maintained in the apiary of the Honeybee Research Institute, Jiangxi Agricultural University, China. To collect age-controlled embryos, mated egg-laying queens were first limited in queen cages for 2–3 h and then placed in the egg- and brood-free areas of their own hives. After 9 hours, the queens were released from the egg-containing honeycombs. After 24 h, 40 embryos per colony were collected and stored together in liquid nitrogen as the first-day embryo samples; the remaining embryos were continuously placed back into their hives. After 48 h, another 40 embryos per colony were collected and stored in liquid nitrogen as the second-day embryo samples; after 72 h, 40 embryos per colony were collected and put into liquid nitrogen as the third-day embryo samples (Additional file 1: Figure S1). In total, we collected nine embryo samples (three biological replicates per day) for RNA-seq.

### Embryo slice making and observation

Fresh *A. cerana* embryos were also collected at 24, 48, and 72 h for the slice preparation. The embryo samples were fixed in 4% paraformaldehyde for 24 h and then transferred into 20% sucrose solution for 48-h dehydration at 4 °C. The dehydrated embryos were embedded in optimal cutting temperature (OCT) compound and then cut into 7-μm sections and placed on glass slides. Each embryo slice was stained by propidium iodide (PI) with a concentration of 50 μg/mL and was immediately observed using a fluorescence microscope (Leica, Germany).

### Next-generation sequencing

Total RNA was extracted from the liquid nitrogen-stored embryo samples using TRIzol reagent according to the manufacturer’s protocol (Invitrogen). The RNA concentration and quantity, RIN, and 28S/18S rRNA ratio of the total RNA samples were determined by using the RNA 6000 Nano Kit on the Agilent 2100 Bioanalyzer (Agilent Technologies, CA, US). The complementary DNA was prepared for each embryo sample using the Illumina mRNA sequencing kit (Illumina, CA, US) and the Clontech SMART cDNA Library Construction Kit (Invitrogen). Libraries were sequenced using the Illumina HiSeq XTen platform (Illumina, CA, US).

### Mapping and quality control of RNA-seq data

Before mapping, raw data were filtered by the following steps: (1) reads with adapters were removed; (2) reads in which unknown bases represented more than 10% of the total sequence were removed; (3) low quality reads, in which the percentage of low-quality bases (quality no more than 10) exceeded 50%, were removed. The remaining reads were defined as “clean reads” and used for subsequent bioinformatics analyses. Quality control on the clean data was performed by the production of a base composition chart and quality distribution chart using FastQC (http://www.bioinformatics.babraham.ac.uk/projects/fastqc/). We used Hisat2 [45] to map clean reads to the *A. cerana* genome assembly version 2.0 (ACSNU-2.0 GCA_001442555.1). Prior to this, exons and splice sites were extracted from the genome annotation GTF files and used to index the genomes. The mapping statistics and the coverage of the gene body by the RNA-seq reads were calculated by using bam_stat.py and geneBody_coverage2.py, respectively, implemented in the RSeQC package [46].

### Transcriptome assembly and annotation

We applied a reference-based transcript assembler, StringTie [47], to assemble the transcripts for each tested embryo sample. The gffcompare program (https://github.com/gpertea/gffcompare) was employed to evaluate the transcript assembles (via comparison with *A. cerana* reference annotation) and to perform the basic tracking of assembled isoforms across multiple RNA-seq experiments. Hmmer2go (https://github.com/sestaton/HMMER2GO) was used to identify the possible open reading frames (ORFs) of the assembled transcripts. We identified putative homologs to the *Drosophila melanogaster* database using BLAST with an E-value threshold of 1e-5, which was implemented in the KOBAS 3.0 online annotation tool [48].

### Differential expression analysis

Transcript abundances were estimated by running StringTie with the -eB options. Read count information was extracted from the above transcript abundance files by prepDE.py for subsequent differential expression analysis. We used the R statistical package DESeq2 [49] from the Bioconductor repository to test for differential expression among the embryo samples from different days.

### GO and KEGG analysis

To determine the functional enrichment of the candidate genes, the fly Gene Ontology database (http://www.geneontology.org) was queried by the ClueGO plugin [50] of Cytoscape using Uniprot ID or EntrezID as the input parameter. The enriched GO terms were characterized according to the default settings. The KEGG pathways were characterized on the KOBAS 3.0 online annotation tool (http://kobas.cbi.pku.edu.cn/annotate.php).

### Quantitative real-time PCR

cDNAs were generated using Reverse Transcriptase kit reagents (Transgen, Beijing, China), according to the manufacturer’s instructions. Fourteen differentially expressed genes were selected for qRT-PCR analysis and *actin 3* was used as an internal control to normalize the data. The primer pairs are provided in Additional file 2: Table S1. Real-time PCR was conducted using an ABI 7500 real-time PCR detection system (ABI, CA, USA). The cycling conditions were as follows: a preliminary cycle at 95 °C for 30 s; 40 cycles of 95 °C for 10 s, 60 °C for 1 min, and 72 °C for 30 s. The specificity of the PCR products was verified by a melting curve analysis for each sample. For each unigene, three biological replicates (with three technical replicates per biological replicate) were performed. The control and target unigenes for each sample were run in the same plate to eliminate interplate variations. The Ct value for each biological replicate was obtained by calculating the arithmetic mean of three technical replicate values. The relative mRNA expression was determined using the comparative 2^-(∆∆Ct)^ method. The statistical analysis of gene expression was performed by analysis of variance (ANOVA) using R version 3.3.3.

## Results

### Embryo stage determination

**Fig. 1:**
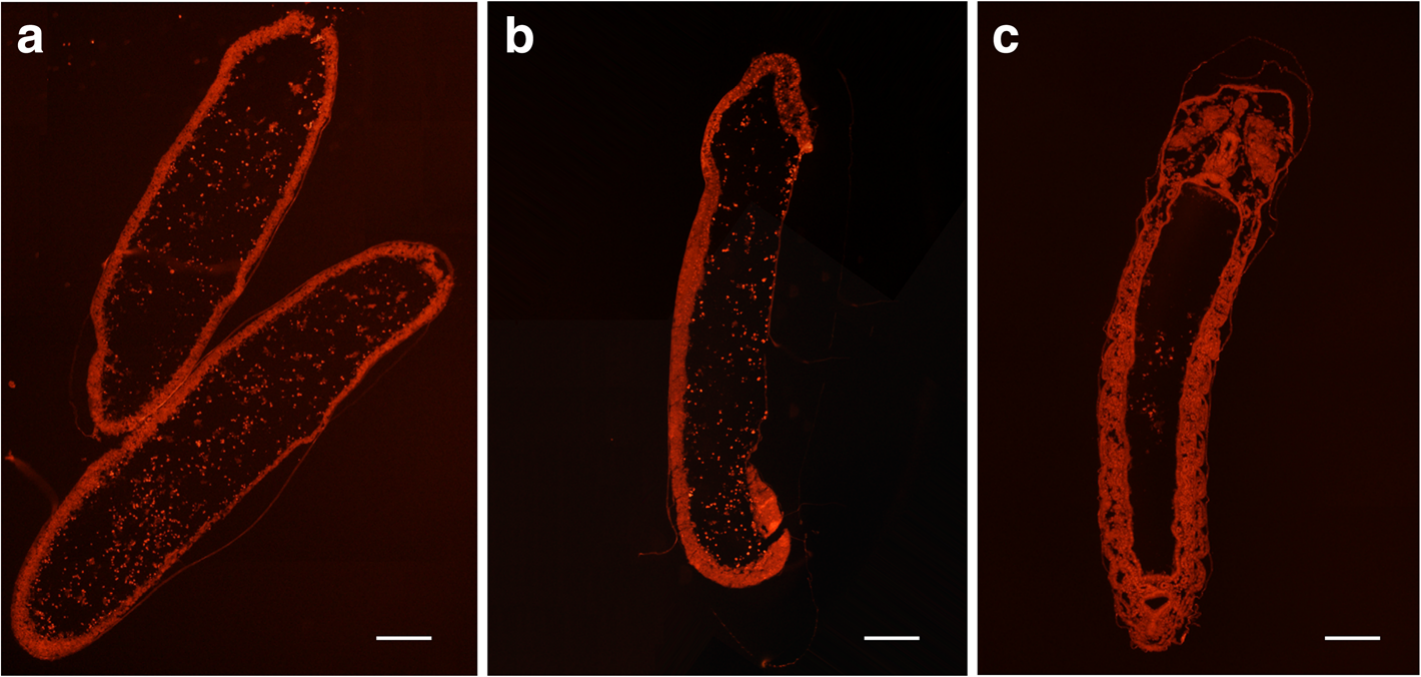
Embryo slices for the Day1, Day2 and Day3 samples. The embryo samples were collected at 24 (Day1), 48 (Day2) and 72 (Day3) hours after the honeybee queens were placed in the egg- and brood-free honeycombs of their own hives. Scale bars = 100 μm

**Fig. 2:**
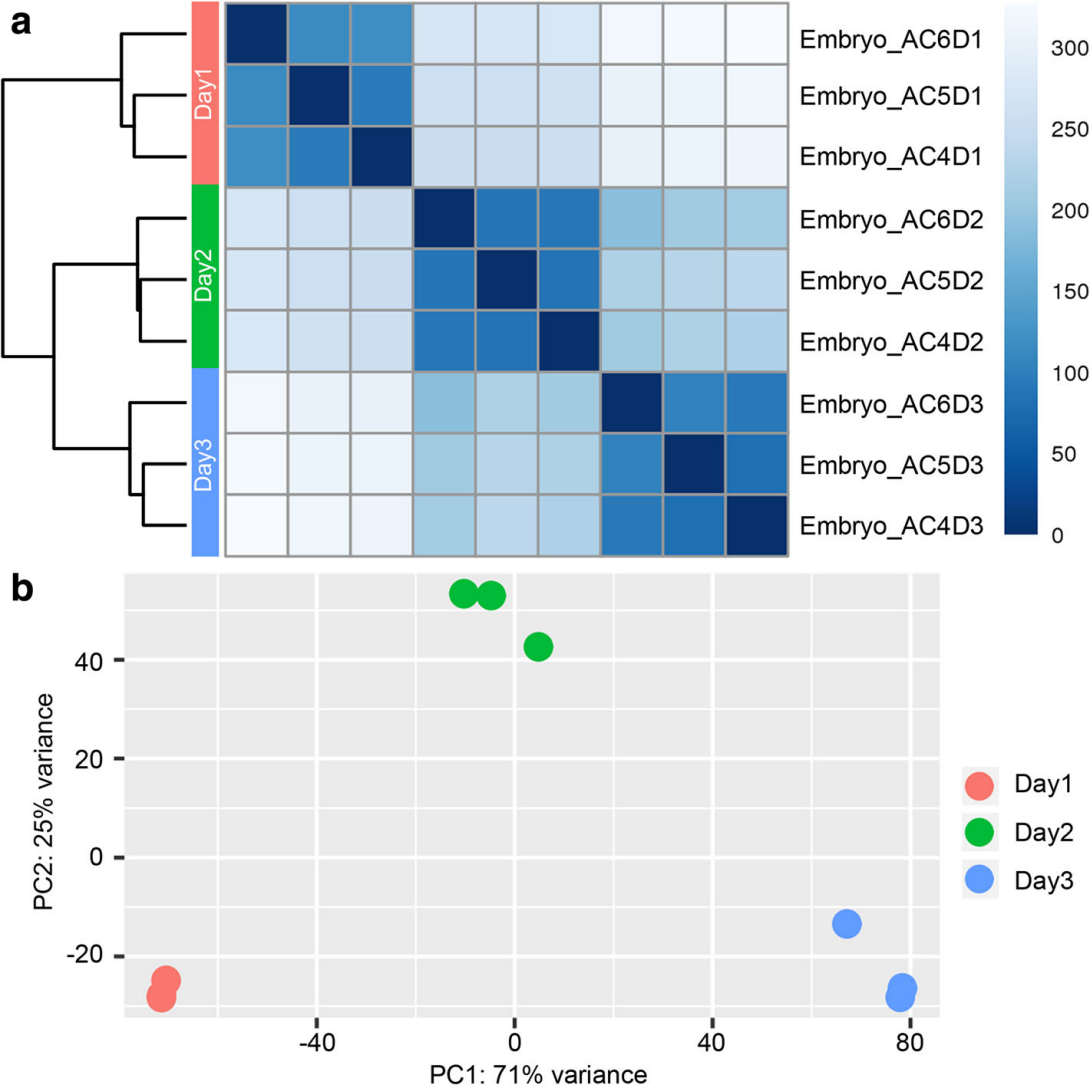
Sample clustering for *A. cerana* in the embryo stage. **a** Heat map and hierarchical clustering of the embryo samples using the whole transcriptome data. **b** Principle component analysis for the embryo samples using the whole transcriptome data

In the embryo slice analysis, large morphological differences were observed among the embryo samples collected at 24, 48 and 72 h after the honeybee queens were placed in the egg- and brood-free honeycombs of their own hives. We determined the developmental stages of our collected samples according to the embryo diagnostic features and stage schemes of the honeybee described by DuPraw [13], Milne, et al. [18] and Cridge, et al. [7]. The Day1 embryo corresponded to approximately late stage four, because blastoderm cells were found along the profile boundary and the inner periplasm extended around the poles (Fig. 1a). The Day2 embryo corresponded to approximately stage eight, as the embryo was at its shortest length and resembled a sausage with an unpaired protuberance at the anterior pole (Fig. 2b). In addition, the Day3 embryo corresponded to stage ten, as the rudimentary morphology of the larva was obviously visible (Fig. 1c).

### Illumina sequencing and mapping statistics

Illumina RNA sequencing generated an average of 65.7 million (M) raw reads per sample. After filtration, an average of 57.3 M clean reads with an accumulated length of 8.6 million bases (Mb) per sample remained for the subsequent transcriptomic analyses. The average Q20 percentage (sequencing error rate, 1%) per sample was 95.3%, and the average GC percentage was 38.3% (Additional file 3: Table S2). With increases in the embryo age, the GC percentage of the clean reads significantly increased from 36.7% (Day1) to 37.6% (Day2, *P* = 9.63 × 10^− 3^) and to 40.6% (Day3, *P* = 5.37 × 10^− 5^), which suggested that the coding sequences in the transcriptomes might increase in length over time. It has been reported that coding sequence length is directly proportional to GC content [51], and the shorter the sequence, the higher the AT bias [52].

The clean reads were mapped to the *A. cerana* reference genome (Assembly ACSNU-2.0) assembled and submitted by Park et al. [37]. The average mapping rate was 74.2%, but 25.8% of the clean reads could not map to the reference genome, which was unlikely to be due to microbial contamination (Additional file 4: Table S3) and indicated that the current *A. cerana* reference genome should be further improved. In the present study, 74.5%, 73.1% and 75.0% of the clean reads from the first-, second- and third-day RNA, respectively, were mapped to the *A. cerana* reference genome. Of these mapped read data, the percentage (77.1%) of properly paired reads from the first day was lower than those from the second day (80.0%, *P* = 7.10 × 10^− 3^) and the third day (82.9%, *P* = 4.41 × 10^− 4^) (Additional file 5: Table S4). Meanwhile, as the age of the embryo increased, the singleton percentage decreased from 16.1% (Day1) to 13.9% (Day2, *P* = 1.05 × 10^− 2^) and 10.6% (Day3, *P* = 6.08 × 10^− 4^). It is also interesting that, as the embryo age increased, the spliced reads increased from 26.7% (Day1) to 28.3% (Day2, *P* = 8.11 × 10^− 3^) and 29.9% (Day3, *P* = 2.76 × 10^− 3^) (Additional file 5: Table S4). These findings suggest that: (1) the transcripts of the first-day embryos were more likely located in regions that are more difficult to be sequenced, possibly due to high GC or AT contents or extensive repeats; (2) larger genes with more intron/exons were expressed in the latest two embryonic development stages; these genes might be involved in morphogenesis and are consistent with the higher GC content of the later-stage embryo transcriptome.

To determine if read coverage was uniform and if there was any 5′/3′ bias, we estimated the gene body coverage for each sample. We found that the gene body coverage was almost symmetric between the 5′ end and 3′ end of the genes, and reads were evenly distributed across the reference genes in each embryo sample (Additional file 6: Figure S2). The results indicated that the RNA quality was high and the randomness of the reads was good, suggesting that the sequencing data of the embryo samples was suitable for further bioinformatic analyses.

### Hierarchical clustering and principal component analysis of samples

To evaluate the consistency of the sample collection and investigate the transcriptomic relationship among the first-, second- and third-day embryos, we performed hierarchical clustering and principal component analysis (PCA) for these samples using the whole gene expression data. Both analyses can provide an overview of similarities and dissimilarities between samples. Before these two analyses, we normalized the gene expression levels among the tested samples by using the regularized log transformation method implemented in the R package of DESeq2 (Additional file 7: Figure S3). We found that the embryo samples collected in the same day were well grouped into the same clusters, and the Day2 samples clustered more closely with the Day3 samples than the Day1 samples (Fig. 2a). In the PCA plot, the embryo samples from the same day were also grouped together (Fig. 2b), which was in agreement with the hierarchical clustering analysis. Both results suggested that the developmental status of the Day2 embryos was closer to that of the Day3 embryos than to that of the Day1 embryos in *A. cerana*.

### Overview of the *A. cerana* embryonic transcriptome

**Fig. 3:**
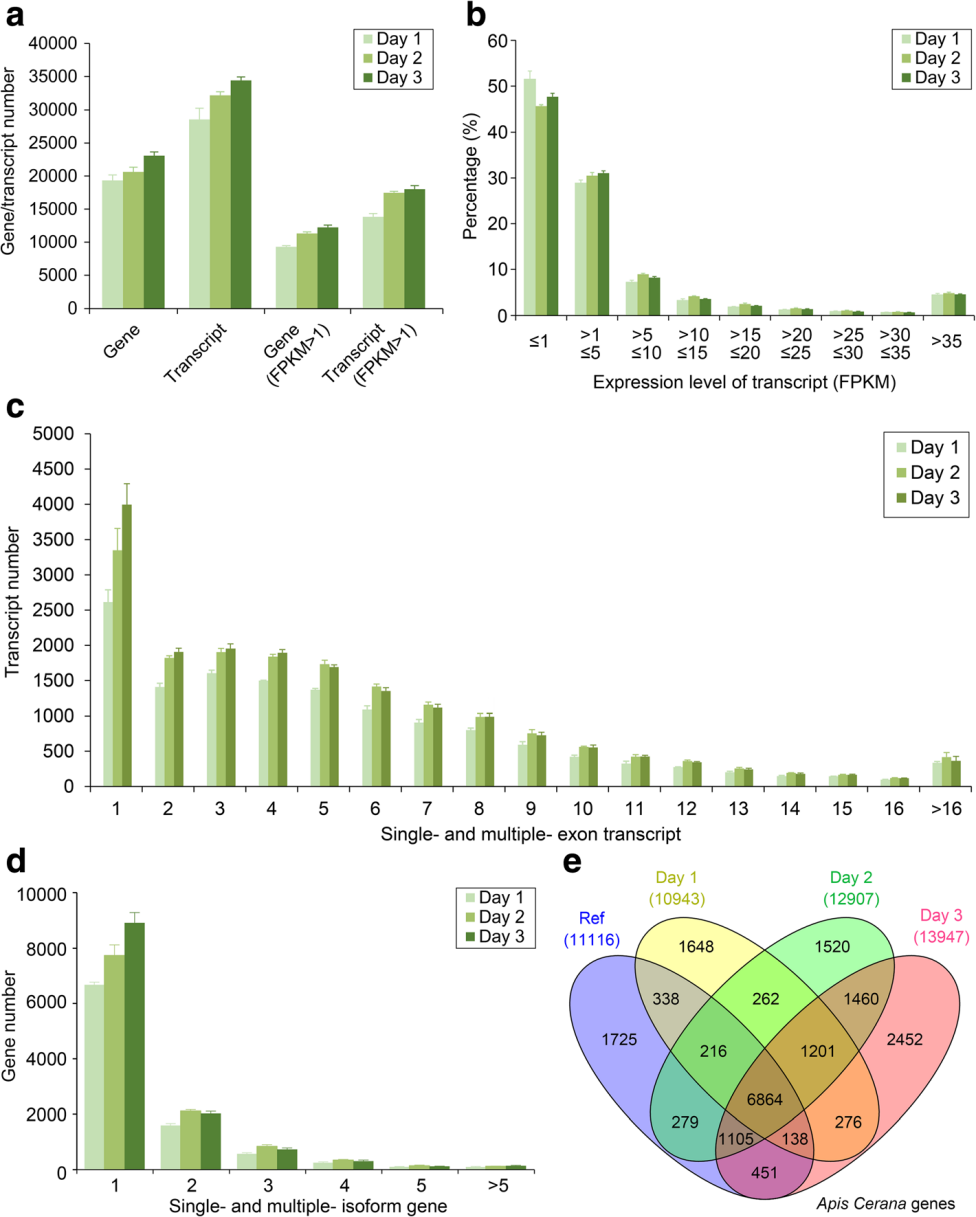
Overview of the embryonic transcriptome. **a** Genes or transcripts detected in the first-, second- and third-day embryos. **b** Transcript expression level in the first-, second- and third-day embryos. **c** Distribution of single- and multi-exon transcripts. **d** Distribution of single- and multi-isoform genes. (E) Comparison among the expressed genes of the first-, second- and third-day embryos and reference genes

We employed StringTie software to reconstruct the embryonic transcriptome for each *A. cerana* embryo sample and to estimate the relative abundances of genes/transcripts in these embryonic transcriptomes. In the present study, we detected an average of 28,561 transcripts per first-day embryo, 32,155 transcripts per second-day embryo and 34,406 transcripts per third-day embryo, which were located on an average of 20,128, 21,441 and 23,876 transcribed loci per sample, respectively (Fig. 3a). When we filtered the low-expression transcripts with the criterion of fragments per kilobase of exon per million fragments mapped (FPKM) > 1, an average of 13,830 transcripts representing 9298 genes per first-day embryo, 17,463 transcripts representing 11,311 genes per second-day embryo, and 18,012 transcripts representing 12,238 genes per third-day embryo remained (Fig. 3a). These results showed that the number of transcripts and transcribed loci generally increased with the increasing embryonic age, indicating that the transcription of embryonic genes became increasingly more active with the developmental time of embryogenesis. The gene expression dynamics during the embryonic development were generally consistent with those of *D. melanogaster* [53].

In the reconstructed transcriptome, 51.5%, 45.7% and 47.7% of transcripts from the first-, second- and third-day embryos showed the low expression level of FPKM ≤1. With increases in the expression abundance, the percentage of the transcripts decreased; for all 3 days, FPKM of ~ 95% transcripts was no more than 35 (Fig. 3b). After filtering the low-expression transcripts with FPKM ≤1, the transcriptomes for all three embryonic ages contained ~ 20% single-exon transcripts and ~ 80% multiexon transcripts. Except for the single-exon transcripts, the number of three-exon transcripts reached a maximum value in the embryonic transcriptomes, and the number of multiexon transcripts decreased with increases in the number of exons (Fig. 3c). In addition, we found that single-isoform genes were much more common than multiple-isoform genes in the embryonic transcriptomes, and the number of single-isoform genes generally increased with embryonic development time (Fig. 3d).

### Discovery of new transcribed regions in the embryonic stage

The transcriptomes (FPKM > 1) of nine tested embryo samples were compared with the annotated *A. cerana* genes deposited in the NCBI GenBank database; the statistical summary of the comparison is shown in Additional file 8: Table S5. Less than half of the transcripts in the embryonic transcriptomes (47.3% for the first day, 46.2% for the second day, 44.7% for the third day) were completely matched with the annotated genes, suggesting that the transcriptomic data identified in this study highly expand the number of known transcribed elements in the *A. cerana* genome and complement the current reference genes to an important extent.

A total of 55,646 transcripts located in 18,284 transcribed regions were detected in the above embryo samples. Of these transcribed regions, 8819 were unannotated during the comparison with the *A. cerana* genes deposited in the NCBI database (Fig. 3c). These unannotated transcript regions harbored 15,292 transcripts. Of these unannotated transcripts, 55.5% had a predicted open reading frame (ORF) greater than 100 amino acids; the remaining transcripts were likely to be non-coding RNAs. We searched these unannotated genes against the *D. melanogaster* protein database using the online KOBAS 3.0 annotation tool [48], and only 364 genes representing 727 transcripts were annotated. The biological functions of the unannotated genes or transcripts need to be further investigated and validated by other experimental methods.

The enriched GO terms and KEGG pathways in these newly annotated genes were determined. We found 7 KEGG pathways (Additional file 9: Table S6), and 112 GO terms were enriched with corrected *P* value < 0.05 (Additional file 10: Table S7) in these genes. The enriched KEGG pathways included phototransduction - fly (dme04745), hippo signaling pathway - fly (dme04391), phagosome (dme04145), ECM-receptor interaction (dme04512), oxidative phosphorylation (dme00190), metabolic pathways (dme01100) and endocytosis (dme04144). GO analysis showed that some key tissue or organ development terms were enriched during embryogenesis, such as tube, epithelium, compound eye, nervous system, muscle structure, wing disc and imaginal disc.

### Core or specific transcriptomes in the development of different day embryos

There were 37,434 transcripts, which represented 8065 genes, that were common to all three embryonic stages of *A. cerana* examined here. Of these transcripts, 89.7% (33,578) were possible coding RNAs with predicted ORFs greater than 100 amino acids. A total of 15% were single-exon transcripts, 85% were multiple-exon transcripts and 46% were multiple-exon multiple isoform transcripts; 68.9% of these transcripts were successfully annotated. We performed GO and KEGG analyses for these annotated core transcripts expressed during embryonic development, and 1098 GO terms (Additional file 11: Table S8) and 50 KEGG pathways (Additional file 12: Table S9) were enriched and had a corrected *P*-value less than 0.05. The top GO biological processes (Table 1) included cellular macromolecule metabolic process (GO:0044260), system development (GO:0048731), cellular developmental process (GO:0048869), gene expression (GO:0010467) and cell differentiation (GO:0030154), while the most significant KEGG pathways (Table 1) were metabolic pathways (dme01100), RNA transport (dme03013), endocytosis (dme04144), spliceosome (dme03040) and protein processing in endoplasmic reticulum (dme04141). These results indicate that these biological processes play key roles in the embryonic development of *A. cerana*, and most of the pathways are likely critical to cellular function in all stages of embryogenesis.

**Table 1:** The top 10 GO terms and KEGG pathways enriched with the common genes in the core transcriptome

ID	GO term or KEGG pathway	*P* Value	Corrected *P* value^a^	Number of Genes
	GO term			
GO:0044260	cellular macromolecule metabolic process	3.50E-221	5.50E-218	2499
GO:0034641	cellular nitrogen compound metabolic process	2.30E-171	3.60E-168	1961
GO:0010467	gene expression	2.00E-158	3.20E-155	1554
GO:0048731	system development	4.00E-157	6.30E-154	1737
GO:0006139	nucleobase-containing compound metabolic process	6.10E-141	9.60E-138	1654
GO:0048869	cellular developmental process	1.20E-140	2.00E-137	1739
GO:0046483	heterocycle metabolic process	1.00E-139	1.50E-136	1689
GO:0006725	cellular aromatic compound metabolic process	1.30E-139	2.10E-136	1727
GO:1901360	organic cyclic compound metabolic process	5.40E-138	8.40E-135	1752
GO:0030154	cell differentiation	7.90E-138	1.20E-134	1673
	KEGG pathway			
dme01100	Metabolic pathways	6.94E-24	1.42E-21	558
dme03013	RNA transport	1.74E-10	1.78E-08	112
dme04144	Endocytosis	3.79E-10	2.57E-08	102
dme03040	Spliceosome	2.10E-09	1.07E-07	100
dme04141	Protein processing in endoplasmic reticulum	7.75E-09	3.16E-07	96
dme00240	Pyrimidine metabolism	1.70E-07	4.96E-06	69
dme04120	Ubiquitin mediated proteolysis	1.70E-07	4.96E-06	76
dme04013	MAPK signaling pathway - fly	6.29E-07	1.60E-05	72
dme03018	RNA degradation	1.29E-06	2.93E-05	50
dme00230	Purine metabolism	3.05E-06	6.23E-05	89

^a^GO Term *P*-values were corrected with the method of Bonferroni step down; KEGG pathway *P*-values were corrected with the method of Benjamini and Hochberg

A total of 1986, 1799 and 2903 genes were specifically expressed in the first-, second- and third-day embryos, respectively (Fig. 3e). We also analyzed these specific transcripts in the embryogenesis stages with GO (Table 2) and KEGG (Additional file 13: Table S10) enrichment analyses. In the first-day embryos, the top GO term was neuropeptide signaling pathway (GO:0007218), which has been reported to regulate synaptic growth in *Drosophila* [54] and indicates that the overrepresented genes might participate in the early development of the nervous system or other related processes in this embryo stage. The second top GO term was response to water (GO:0009415), which indicated that water utilization and/or water responses are important in early embryonic development [55]. Surprisingly, the eye-antennal disc development (GO:0035214), morphogenesis (GO:0007455), leg disc development (GO:0007478) and pigment metabolic process (GO:0042440) biological processes were detected in the transcripts from the first day of embryo development. Possibly, the eye- and leg-disc development processes were initialized at the early embryonic stage of *A. cerana*. For Day2, only seven GO terms were found, which mainly included the organic acid biosynthetic process, olfactory behavior, photoreceptor cell axon guidance, tRNA processing, and ion transport terms. For Day3, the ion transport and homeostasis biological processes were found frequently, indicating that ion transport and homeostasis are important events during the third day of the development of *A. cerana* embryos.

**Table 2:** GO terms enriched with the specific genes expressed in the first-, second- and third-day embryos

Day	GOID	GOTerm	Term *P* Value	Term *P* Value Corrected^a^	Number of genes	Associated Genes Found
1	GO:0007218	neuropeptide signaling pathway	5.40E-06	1.00E-04	6	[CCAP-R, CG33639, CapaR, ETHR, FMRFaR, Lkr]
1	GO:0009415	response to water	1.50E-05	2.80E-04	3	[CapaR, Poxn, ppk28]
1	GO:0042440	pigment metabolic process	1.90E-04	3.30E-03	6	[CG1885, Cdk5alpha, Itgbn, cd, dl, yellow-d2]
1	GO:0035214	eye-antennal disc development	8.20E-03	8.20E-03	3	[Poxn, bab1, dl]
1	GO:0006582	melanin metabolic process	6.90E-04	1.10E-02	4	[Cdk5alpha, Itgbn, dl, yellow-d2]
1	GO:0001101	response to acid chemical	1.00E-03	1.50E-02	3	[CapaR, Poxn, ppk28]
1	GO:0040012	regulation of locomotion	7.90E-03	1.50E-02	3	[Hr51, Hrs, Ten-m]
1	GO:0072507	divalent inorganic cation homeostasis	5.10E-03	1.50E-02	3	[FMRFaR, Lkr, trpl]
1	GO:0010035	response to inorganic substance	4.60E-03	1.80E-02	3	[CapaR, Poxn, ppk28]
1	GO:0018958	phenol-containing compound metabolic process	1.50E-03	1.90E-02	4	[Cdk5alpha, Itgbn, dl, yellow-d2]
1	GO:0071214	cellular response to abiotic stimulus	1.30E-03	1.90E-02	4	[Arr1, CapaR, Gat, ppk28]
1	GO:0007478	leg disc morphogenesis	1.80E-03	2.20E-02	4	[Poxn, bab1, dl, trio]
1	GO:0042551	neuron maturation	4.40E-03	2.20E-02	3	[Drice, Hr51, Hrs]
1	GO:0006874	cellular calcium ion homeostasis	2.10E-03	2.30E-02	3	[FMRFaR, Lkr, trpl]
1	GO:0007455	eye-antennal disc morphogenesis	2.10E-03	2.30E-02	3	[Poxn, bab1, dl]
1	GO:0016322	neuron remodeling	4.00E-03	2.40E-02	3	[Drice, Hr51, Hrs]
1	GO:0055074	calcium ion homeostasis	2.40E-03	2.40E-02	3	[FMRFaR, Lkr, trpl]
1	GO:0072503	cellular divalent inorganic cation homeostasis	3.80E-03	2.60E-02	3	[FMRFaR, Lkr, trpl]
1	GO:0035006	melanization defense response	3.40E-03	2.70E-02	3	[Cdk5alpha, Itgbn, dl]
1	GO:0009755	hormone-mediated signaling pathway	3.20E-03	2.90E-02	3	[ETHR, Hr51, Lkr]
2	GO:0016053	organic acid biosynthetic process	7.90E-04	5.50E-03	5	[Baldspot, CG5278, Dh44-R2, Oat, b]
2	GO:0042048	olfactory behavior	2.00E-03	1.00E-02	5	[CG14509, Calr, NaCP60E, TkR99D, prt]
2	GO:0072499	photoreceptor cell axon guidance	1.70E-03	1.00E-02	3	[Nrk, gogo, jbug]
2	GO:0008033	tRNA processing	1.60E-02	1.60E-02	3	[CG10495, CG15618, CG4611]
2	GO:0046394	carboxylic acid biosynthetic process	4.50E-03	1.80E-02	4	[Baldspot, CG5278, Dh44-R2, Oat]
2	GO:0098656	anion transmembrane transport	1.50E-02	3.10E-02	3	[Best1, Indy, NaPi-T]
2	GO:0043269	regulation of ion transport	1.00E-02	3.20E-02	3	[Best1, NaCP60E, Rgk1]
3	GO:0006811	ion transport	7.00E-07	5.30E-05	24	[CG10960, CG14507, CG31028, CG31547, CG3690, CG42269, CG5002, CG5621, CG6125, CG6356, CG8249, Ca-alpha1T, Ctr1A, Gat, HisCl1, Mco1, Nckx30C, Nmdar1, RyR, Trpgamma, Zip42C.1, Zip89B, nAChRbeta1, para]
3	GO:0055080	cation homeostasis	1.30E-06	9.90E-05	11	[CG5002, CG6125, Ctr1A, Dat, Mco1, Nckx30C, Nmdar1, RyR, Trpgamma, Zip42C.1, norpA]
3	GO:0098771	inorganic ion homeostasis	1.60E-06	1.20E-04	11	[CG5002, CG6125, Ctr1A, Dat, Mco1, Nckx30C, Nmdar1, RyR, Trpgamma, Zip42C.1, norpA]
3	GO:0050801	ion homeostasis	2.30E-06	1.70E-04	11	[CG5002, CG6125, Ctr1A, Dat, Mco1, Nckx30C, Nmdar1, RyR, Trpgamma, Zip42C.1, norpA]
3	GO:0055065	metal ion homeostasis	3.80E-06	2.70E-04	9	[Ctr1A, Dat, Mco1, Nckx30C, Nmdar1, RyR, Trpgamma, Zip42C.1, norpA]
3	GO:0009072	aromatic amino acid family metabolic process	7.70E-06	5.50E-04	5	[CG1461, Trh, hgo, v, y]
3	GO:0072507	divalent inorganic cation homeostasis	1.20E-05	8.40E-04	7	[Dat, Nckx30C, Nmdar1, RyR, Trpgamma, Zip42C.1, norpA]
3	GO:0030003	cellular cation homeostasis	1.40E-05	1.00E-03	9	[CG5002, CG6125, Ctr1A, Dat, Mco1, Nckx30C, RyR, Trpgamma, norpA]
3	GO:0006873	cellular ion homeostasis	2.10E-05	1.40E-03	9	[CG5002, CG6125, Ctr1A, Dat, Mco1, Nckx30C, RyR, Trpgamma, norpA]
3	GO:0055074	calcium ion homeostasis	2.70E-05	1.80E-03	6	[Dat, Nckx30C, Nmdar1, RyR, Trpgamma, norpA]
3	GO:0098660	inorganic ion transmembrane transport	3.30E-05	2.20E-03	14	[CG31028, CG31547, CG5002, CG6125, Ca-alpha1T, Ctr1A, HisCl1, Mco1, Nckx30C, RyR, Trpgamma, Zip42C.1, Zip89B, para]
3	GO:0055082	cellular chemical homeostasis	4.90E-05	3.20E-03	9	[CG5002, CG6125, Ctr1A, Dat, Mco1, Nckx30C, RyR, Trpgamma, norpA]
3	GO:0006875	cellular metal ion homeostasis	5.70E-05	3.60E-03	7	[Ctr1A, Dat, Mco1, Nckx30C, RyR, Trpgamma, norpA]
3	GO:0000041	transition metal ion transport	1.20E-04	7.80E-03	5	[Ctr1A, Mco1, Trpgamma, Zip42C.1, Zip89B]
3	GO:1901565	organonitrogen compound catabolic process	1.30E-04	8.30E-03	9	[CG5418, CG8129, CG9380, Dat, GLS, PGRP-LB, hgo, v, verm]
3	GO:0048878	chemical homeostasis	1.40E-04	8.90E-03	11	[CG5002, CG6125, Ctr1A, Dat, Mco1, Nckx30C, Nmdar1, RyR, Trpgamma, Zip42C.1, norpA]
3	GO:0019725	cellular homeostasis	1.90E-04	1.10E-02	11	[CG5002, CG6125, CG6888, Ctr1A, Dat, Mco1, Nckx30C, RyR, Trpgamma, norpA, tn]
3	GO:0006874	cellular calcium ion homeostasis	2.50E-04	1.50E-02	5	[Dat, Nckx30C, RyR, Trpgamma, norpA]
3	GO:0042430	indole-containing compound metabolic process	3.30E-04	1.90E-02	3	[Dat, Trh, v]
3	GO:0044550	secondary metabolite biosynthetic process	3.80E-04	2.10E-02	5	[Elo68alpha, v, y, yellow-c, yellow-e2]
3	GO:0046189	phenol-containing compound biosynthetic process	5.10E-04	2.80E-02	4	[Trh, y, yellow-c, yellow-e2]
3	GO:1901606	alpha-amino acid catabolic process	5.10E-04	2.80E-02	4	[CG8129, GLS, hgo, v]
3	GO:0070838	divalent metal ion transport	6.40E-04	3.50E-02	6	[Ca-alpha1T, Nckx30C, RyR, Trpgamma, Zip42C.1, Zip89B]
3	GO:0072511	divalent inorganic cation transport	6.40E-04	3.50E-02	6	[Ca-alpha1T, Nckx30C, RyR, Trpgamma, Zip42C.1, Zip89B]
3	GO:0072503	cellular divalent inorganic cation homeostasis	6.60E-04	3.60E-02	5	[Dat, Nckx30C, RyR, Trpgamma, norpA]
3	GO:0006820	anion transport	7.90E-04	4.20E-02	8	[CG14507, CG31547, CG3690, CG5002, CG6125, CG6356, Gat, HisCl1]
3	GO:0015711	organic anion transport	8.30E-04	4.30E-02	6	[CG14507, CG3690, CG5002, CG6125, CG6356, Gat]

^a^GO Term *P*-values were corrected with the method of Bonferroni step down

### Differentially expressed genes and their expression patterns

**Fig. 4:**
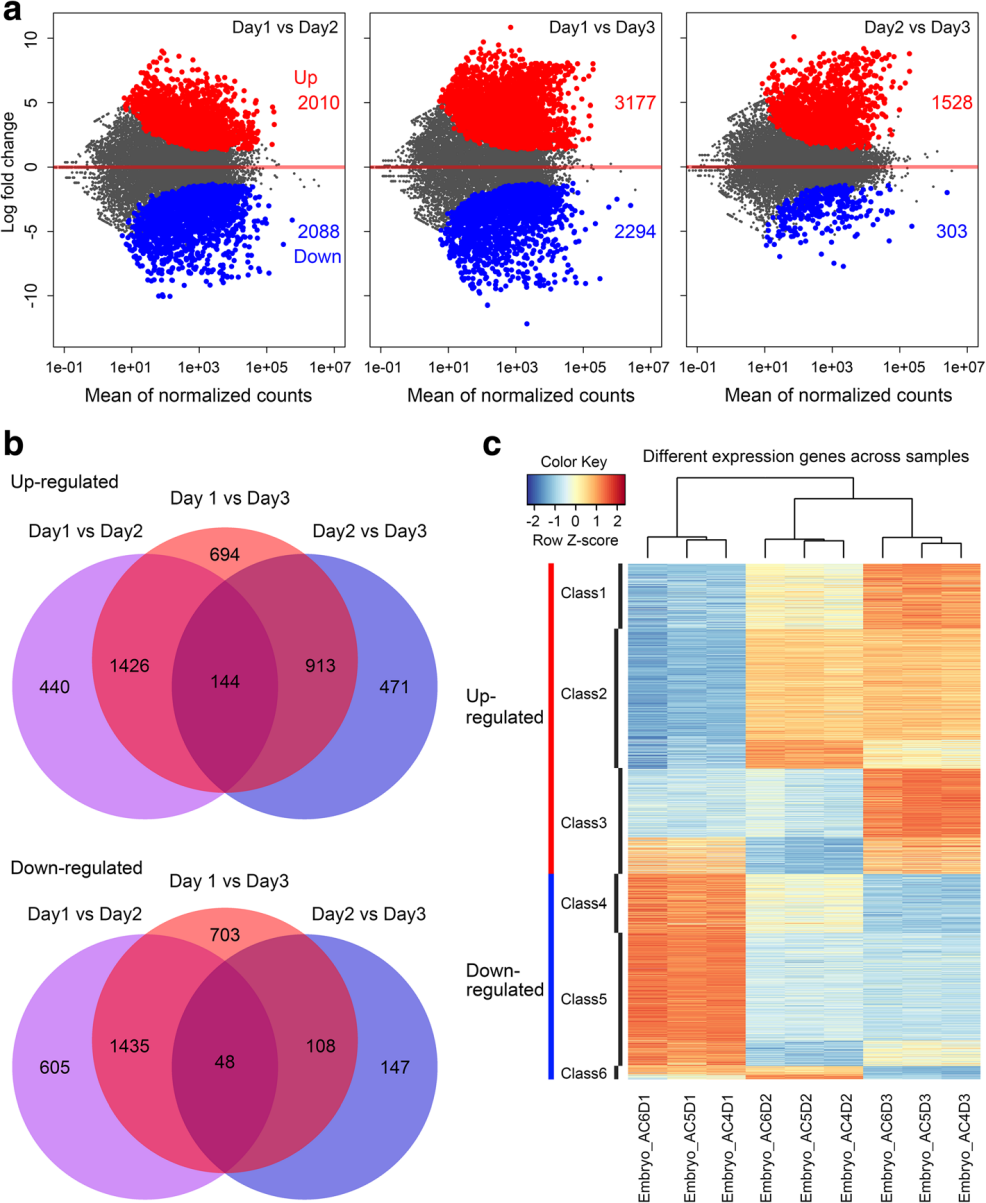
Differential gene expression among the first-, second- and third-day embryos. **a** Pairwise comparison among the differential expressed genes of the first-, second- and third-day embryos. **b** Up- and down-regulated genes of the first-, second- and third-day embryos. **c** Hierarchical clustering and heat map for all embryo samples using differentially expressed gene data

In the present study, we identified 4088 upregulated and 3046 downregulated differentially expressed genes (DEGs) among the three ages of embryo samples. Of these DEGs, 2010, 3177 and 1528 genes were upregulated and 2088, 2294 and 303 genes were downregulated from Day1 to Day2, from Day1 to Day3 and from Day2 to Day3, respectively (Fig. 4a). Generally, the total number of upregulated genes was higher than that of downregulated genes, especially for the DEGs from Day2 to Day3. There were much less DEGs from Day2 to Day3 than from Day1 to Day2, indicating that the change during development between Day1 and Day2 was much larger than that between Day2 and Day3 (Fig. 4a and b). This result is consistent with the sample clustering using the full gene set (Fig. 2a) or the DEG set (Fig. 4c).

We further classified these DEGs into 6 groups based on the expression patterns (Additional file 14: Figure S4): (1) Class one, continuous upregulated; (2) Class two, upregulated from Day1 to Day2, but not significantly different between Day2 and Day3; (3) Class three, upregulated from Day2 to Day3, but not significantly different between Day1 and Day2; (4) Class four, continuously downregulated; (5) Class five, downregulated from Day1 to Day2, but not significantly different between Day2 and Day3; (6) Class six, downregulated from Day2 to Day3, but not significantly different between Day1 and Day2. We found the largest DEG number in Class 2 (upregulated from Day1 to Day2), 1781, and the second largest DEG number was in Class 5 (downregulated from Day1 to Day2); these results also suggested there were many gene expression changes from Day1 to Day2, and the development between Day1 and Day2 was highly dynamic. We selected 14 DEGs from different classes of the above expression patterns, most of which had a known biological function. We employed qRT-PCR to validate their gene expression. The mRNA expression trends of the qRT-PCR-tested genes were consistent with those detected by RNA-seq (Additional file 15: Figure S5).

GO analysis (Additional file 16: Table S11) showed that more than one hundred continuously upregulated genes participated in the biological processes of multicellular organism development, system development, cell differentiation and development, and anatomical structure morphogenesis, and hundreds of continuously downregulated genes took part in the regulation of cellular and biosynthetic processes, macromolecule modification and metabolic processes, and other biological processes. In the Class 2, 3, 5 and 6 DEGs, the top GO terms again referred to system development, animal organ development and multicellular organism development, which indicated that, during the entire embryonic development, many differentially expressed genes are involved in the key biological processes of system, organ or multicellular organism development. Notably, in Class 2, GO terms related to epithelium, tube and neuron development were enriched, while in Class 3, GO terms related to muscle development were repeatedly observed. These findings indicated that neuronal and epithelial development occurred earlier than muscle development during honeybee embryogenesis.

In addition, many core genes were differentially expressed in the three *A. cerana* embryo ages, indicating that these core genes are regulated throughout embryonic development. We found that 203 GO terms specifically overlapped with those enriched by the downregulated genes, 43 GO terms specifically overlapped with those enriched by the upregulated genes and 110 GO terms overlapped simultaneously with the terms enriched by both the up- and downregulated genes (Additional file 11: Table S8).

### Comparison with previous studies on honeybee embryonic development

Previously, many key genes regulating honeybee embryonic development have been studied [24–26, 56–59]. Here, we found most of these genes were differentially expressed in the three studied ages of *A. cerana* embryos. For example, we detected *empty-spiracle* (*ems*), a conserved head-patterning gene, in our Day1, Day2 and Day3 embryo transcriptomes, which corresponded to late stage four, stage eight and stage ten, respectively. Its expression abundance was highest in Day2 and was lowest in Day1 (Additional file 17: Figure S6). This expression pattern was highly consistent with the results of in situ hybridization [56].

**Fig. 5:**
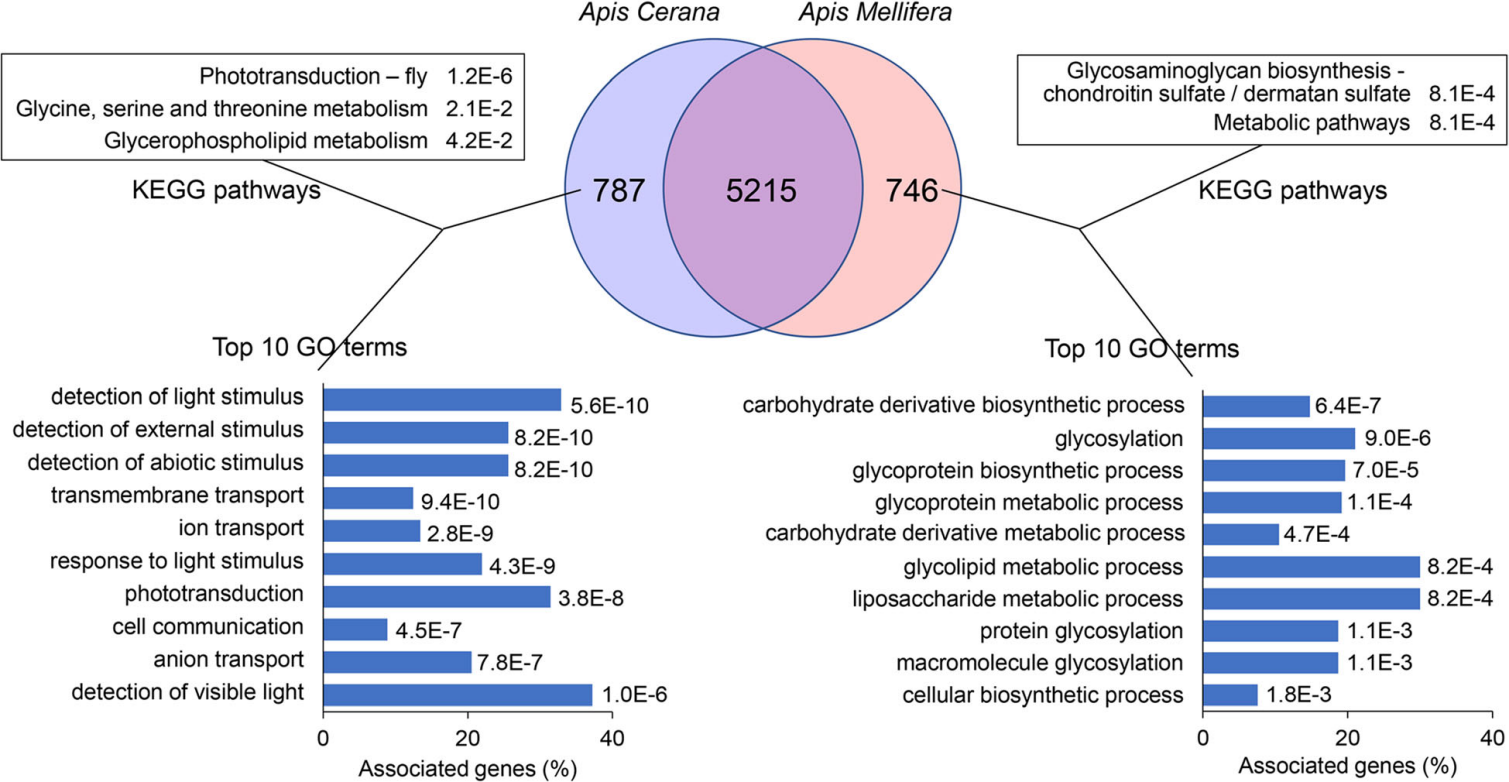
Comparison between annotated genes in the first-day embryo transcriptome of *A. cerana* and *A. mellifera*. Venn diagram shows the number of specific and common genes expressed in *A. cerana* and *A. mellifera*. All KEGG pathways listed have enrichment *P* values < 0.05, and the top 10 GO terms are highlighted (for the complete list of GO terms, refer to Additional File 18: Table S12). Corrected *P*-values were shown on the right flanking sites

Recently, the early zygotic transcriptions of European honeybee (*A. mellifera*) at 0–2, 0–6 and 18–24 h were investigated [38]. Among the embryonic samples of this study, the sample collected at 18–24 h was very close in age to our first-day embryo samples. Therefore, we downloaded the raw RNA-seq data of the 18–24 h embryo sample and compared the data with our transcriptome for the first-day *A. cerana* embryos. The descriptive statistics of the RNA-seq, mapping and annotation are listed in Additional file 18: Table S12. In general, the quality of our RNA-seq data was higher, the data size was about six times larger than that of the downloaded data, and more transcripts were identified in our *A. cerana* embryos. We annotated both the *A. mellifera* and *A. cerana* transcripts against the *D. melanogaster* protein database using the KOBAS 3.0 online annotation tool [48]. In total, 5961 *A. mellifera* and 6002 *A. cerana* genes were annotated. We found 86.9% (5215) of the annotated *A. cerana* genes were shared by the *A. mellifera* first-day embryo; in addition, 787 genes were specifically expressed in *A. cerana*, while 746 genes were specifically expressed in *A. mellifera* (Fig. 5). Among the common genes, 98% (5116 genes) were core genes expressed in all *A. cerana* embryos throughout the entire embryonic development.

GO and KEGG analyses were performed to identify the biological processes and pathways of the specific genes expressed in *A. cerana* and *A. mellifera*. A total of 79 and 28 GO terms for the first-day *A. cerana* and *A. mellifera* embryos had a corrected enrichment *P*-value less than 0.05 (Additional file 19: Table S13), while three and two KEGG pathways of *A. cerana* and *A. mellifera*, respectively, passed the same filtering criterion (Fig. 5). Notably, in *A. cerana*, the most significant KEGG pathway was phototransduction - fly (dme04745), and six of the ten top GO terms were related to the light detection biological process, namely, the detection of light stimulus (GO:0009583), detection of external stimulus (GO:0009581), detection of abiotic stimulus (GO:0009582), response to light stimulus (GO:0009416), phototransduction (GO:0007602) and detection of visible light (GO:0009584) terms. These findings suggest that the gene expression related to light detection in the early embryo stage of *A. cerana* largely differs from that of *A. mellifera*, which is valuable information for further investigations. On the other hand, in *A. mellifera*, the top KEGG pathway (Fig. 5) was glycosaminoglycan biosynthesis - chondroitin sulfate / dermatan sulfate (dme00532). The top 10 GO terms were associated with the glycosylation and glycoprotein metabolic process (Fig. 5). Chondroitin sulfate proteoglycan is an important component of the extracellular matrix in the central nervous system, and it takes part in cell migration in the central nervous system [60]. Therefore, the development of the central nervous system in *A. mellifera* might occur earlier than that in *A. cerana*.

Another study on the embryo development of the European honeybee (*A. mellifera*) was recently performed by Fang et al. [41]. They employed mass spectrometry-based proteomics to investigate the proteomic alterations of honeybee embryogenesis for ages of 24, 48 and 72 h. In our *A. cerana* transcriptome analyses, the sample collection and analysis strategies were similar to those of Fang et al. We found that the clustering of the embryo samples was consistent with their clustering results for *A. mellifera*, in which the second- and third-day samples were close to each other and clustered together, and the first-day samples were grouped into another cluster. Fang, et al. successfully annotated 845, 1078 and 1116 proteins with the KEGG database at 24, 48 and 72 h, respectively. In contrast, in the present study, we identified 6002, 6677 and 6831 annotated proteins, respectively. Far more common metabolic pathways were significantly enriched in the embryos during the three stages, and more genes were included in each enriched metabolic pathway in our transcriptomic data. For example, in the proteomic analysis, 16 proteins were enriched in the glycolysis/gluconeogenesis pathway, while in our transcriptome, 27 genes were detected and enriched in the same pathway, including the 16 proteins identified by Fang et al. (Additional file 20: Figure S7).

## Discussion

Embryogenesis is an elementary and fundamental stage of insect development, and it plays an important role in supporting the developmental process of the whole life cycle. Before the omics era, honeybee embryogenesis was characterized through morphological observations using light microscopy [17, 61] and/or scanning electron microscopy [12, 62], and rich morphological data and accurate staging scheme of honeybee embryogenesis were provided [13, 17, 18, 63]. In addition, many key developmental genes have been studied, and their biological functions have been characterized in patterning the honeybee embryo [24–26, 56–59]. These works have provided us with a great deal of important developmental knowledge about honeybee embryogenesis. However, the studies on honeybee embryogenesis have mainly focused on *A. mellifera*, including the proteomic characterization of *A. mellifera* embryogenesis [41, 42] and the early zygotic transcriptome analysis of haploid and diploid honeybee embryonic cleavage [38]. Currently, proteomics cannot yield as much developmental information as transcriptomics in embryo or tissue functional research. Overviews of embryonic transcriptomes and the dynamic regulation of embryonic transcription are still largely uncharacterized in honeybee species, especially in *A. cerana*. To our knowledge, this study was the first to employ transcriptomics to study the embryonic development of *A. cerana*.

The embryonic developmental period of both *A. cerana* and *A. mellifera* is 3 days (72 h). We sampled the first-, second- and third-day embryos of *A. cerana* to perform the transcriptomics research. According to the staging scheme of honeybee embryos and the observed morphological characters, these Day1, Day2 and Day3 embryos likely corresponded to the late stage four, stage eight and stage ten developmental stages, respectively. We analyzed nine deep-sequenced RNA libraries from these age-controlled embryos of *A. cerana*. In total, more than 500 million clean reads were mapped to the *A. cerana* database (ACSNU-2.0). The hierarchical clustering and principal component analysis results for the samples showed that the sample homogeneity was good, and these time-collected samples could well represent the three ages of *A. cerana* embryos. Additionally, the analyses of gene body coverage showed that the qualities of the extracted RNA and RNA-seq data were high, and the RNA-seq data were suitable for further transcriptomic analyses. In addition, the qRT-PCR results validated the reliability of the transcriptome data and the DEG analyses. In the present study, we identified many new elements, including thousands of genes, coding and non-coding transcripts, exons and splicing events. These data substantially expand the number of known transcribed elements in the *A. cerana* genome and may be useful for comparative studies between honeybee species.

The overall mRNA expression generally increased with the developmental time of the *A. cerana* embryos, which was similar to the case of *Drosophila* [53]. Meanwhile, the gene expression differences between the first- and third-day embryos were the most significant, and the expression pattern of the second day was much closer to that of the third day than to that of the first day. That is, the mRNA expression changes from the first day to the second day were greater than those from the second day to the third day. This transcriptomic pattern was consistent with the proteomic changes of *A. mellifera* revealed by proteomics [41], suggesting that the developmental patterns of both *A. mellifera* and *A. cerana* were similar from the overall perspective of embryogenesis.

In this study, we detected a total of 18,284 genes expressed in the *A. cerana* embryos, of which 44.5% were core genes commonly expressed throughout the entire period of embryonic development. These core genes participated in a series of important biological processes and pathways, and many core genes were differentially expressed in the three studied ages (approximately 24, 48 and 72 h) of the *A. cerana* embryos. The downregulated GO terms included many basic biological processes, such as RNA processing and splicing, macromolecule modification, cellular component assembly, oogenesis and oocyte development, mitotic cell cycle process, axis specification, and others. Most of the downregulated GO terms were expressed in the early embryogenesis of *A. cerana*. We also found the morphogenesis of embryonic epithelium, embryonic axis specification and neuroblast proliferation terms were downregulated, indicating these biological processes occur in the early period of *A. cerana* embryonic development. These developmental events also occurred in the early period (stages 7–9) of *Drosophila* embryonic development [64]. The upregulated GO terms were involved in cell fate determination and many tissue or system developments, including respiratory system, circulatory system, muscle, gland, brain, head, and leg disc developments. These developmental events were consistent with those in the late period (stages 15–17) of *Drosophila* embryonic development [64]. The GO terms that simultaneously enriched by both the up- and downregulated genes mainly participated in segmentation, nervous system development, epithelial tube and wing disc development and eye morphogenesis, which usually develop during stages 11–13 of *Drosophila* embryogenesis [64, 65].

A total of 10,943 genes were expressed in the first day of *A. cerana* embryonic development, of which 1986 were specifically expressed in the first day, not in the second and third day. These specifically expressed genes were involved in neuropeptide and hormone-mediated signaling pathways, pigment and melanin metabolic processes, calcium ion homeostasis, neuronal maturation, eye-antennal and leg disc developments, responses to water, and other processes. For the second-day *A. cerana* embryo, a total of 12,907 genes were expressed. Among these expressed genes, 1799 were specifically expressed in the second day. These genes were involved in the organic acid biosynthetic process, olfactory behavior, photoreceptor cell axon guidance, anion transmembrane transport and ion transport regulation. These results indicate that eye morphogenesis continued and olfactory tissue might be developed in stage eight of *A. cerana* embryonic development. In addition, organic acids might play an important role in this stage. A total of 13,947 genes were expressed on the third day of *A. cerana* embryonic development. Among these genes, 2903 were specifically expressed in the third day and not in the first and second day. The ion transport and ion homeostasis biological processes were enriched with these specifically-expressed genes. Ion channels, pumps and exchangers are also useful markers for cell fate determination, as electrical stimulation modulates fate determination of differentiating embryonic stem cells [66]. Therefore, ion transport and homeostasis might play important roles in cell differentiation and cell fate determination during honeybee embryogenesis. The specific genes mentioned above and their enriched biological pathways require experiments for validation.

We compared *A. cerana* and *A. mellifera* embryogenesis; the overall trends in the embryonic development of these species were similar. The developmental status between the second day and the third day was closer than that between the first day and the second or third day in both *A. cerana* (Fig. 1b) and *A. mellifera* [41]. In addition, we compared the transcriptomes of the first-day embryos of *A. cerana* and *A. mellifera*. We found more than 80% of the expressed genes were shared in both honeybee species. Notably, the biological processes related to light detection and responses, e.g., the response to and detection of light stimuli, and phototransduction were repeatedly enriched in the specifically expressed genes of *A. cerana* during the first day of embryogenesis. On the other hand, in *A. mellifera*, glycosylation and glycoprotein metabolic processes were enriched, and glycosaminoglycan biosynthesis - chondroitin sulfate/dermatan sulfate terms were detected in the specifically expressed genes of *A. mellifera* during the first day of embryogenesis. Chondroitin sulfate proteoglycan is an important component of the extracellular matrix in the central nervous system and plays essential roles in cell migration in the central nervous system [60]. These findings suggest that: (1) the eye development of *A. cerana* might be highly different from that of *A. mellifera*, and (2) the development of the central nervous system in *A. mellifera* possibly occurs earlier than that in *A. cerana*. In the future, embryos of *A. cerana* and *A. mellifera* at different time periods should be compared to better understand honeybee embryogenesis and differences between species. This could provide a developmental basis for the study of adult honeybee morphogenesis and behavior.

## Conclusions

We applied high-quality RNA-seq to analyze the dynamic expression of the *A. cerana* transcriptome during embryogenesis. A total of 18,284 genes were identified, including 8065 core genes and 8819 newly annotated genes, which extended the existing known gene database of *A. cerana*. We found that the continually downregulated genes were highly involved in cellular, biosynthetic and metabolic processes, gene expression and protein localization, cell cycle and macromolecule modification. In addition, the consistently upregulated genes mainly participated in cell development and differentiation, tissue, organ and system development, anatomical structure morphogenesis, and learning and cognition. There were two notable differences in the first-day embryonic development of *A. cerana* and *A. mellifera*: (1) eye development was noticeably different between both honeybees; and (2) the central nervous system development of *A. mellifera* might occur earlier than that of *A. cerana*. The molecular mechanism underlying both differences is worthy of further study.

## Additional files

Additional file 1: **Figure S1.** Collection of the *A. cerana.* embryo samples. (TIFF 214 kb)

Additional file 2: **Table S1.** Differentially expressed genes and their primers for qRT-PCR. (DOCX 19 kb)

Additional file 3: **Table S2.** RNA-seq statistics of collected embryo samples. (DOCX 14 kb)

Additional file 4: **Table S3.** The number of aligned reads in a search of the NCBI nt database using 10,000 randomly selected unmapped reads. (XLSX 15 kb)

Additional file 5: **Table S4.** Mapping statistics of RNA-seq data. (DOCX 14 kb)

Additional file 6: **Figure S2.** Gene body coverage of all the embryo samples. These results show that the RNA-seq libraries and RNA-seq data of our embryo samples were good enough and were suitable for the subsequent bioinformatic analyses. (TIFF 965 kb)

Additional file 7: **Figure S3.** Unnormalized and normalized transcript expression levels of our embryo samples and seven other tissues of *A. cerana.* downloaded from the NCBI database. (TIFF 410 kb)

Additional file 8: **Table S5.** Summary of the embryonic transcriptomes (FPKM > 1) and comparison with the annotated *A. cerana.* reference genes. (DOCX 17 kb)

Additional file 9: **Table S6.** KEGG pathways enriched with novel genes identified in the embryonic development. (DOCX 14 kb)

Additional file 10: **Table S7.** GO terms enriched with novel genes identified in the embryonic development. (XLSX 22 kb)

Additional file 11: **Table S8.** GO terms enriched by the core transcriptome in the embryonic development. (XLSX 509 kb)

Additional file 12: **Table S9.** KEGG pathways enriched by the core transcriptome during the embryonic development. (XLSX 20 kb)

Additional file 13: **Table S10.** KEGG pathways enriched with the specific genes expressed in the first-, second- and third-day embryos. (XLS 9 kb)

Additional file 14: **Figure S4.** Expression pattern of differentially expressed genes. These differentially expressed genes were categorized into six expression model classes. (TIFF 345 kb)

Additional file 15: **Figure S5.** Comparison between the RNA-seq and RT-qPCR results for 14 differential expressed genes identified by the R statistical package DEseq2. Most of the differential expressed genes were validated by ANOVA, and their expression patterns based on qRT-PCR were generally consistent with the ones based on RNA-seq. (TIFF 170 kb)

Additional file 16: **Table S11.** GO terms enriched by the six different classes of DEGs during the embryonic development. (XLSX 149 kb)

Additional file 17: **Figure S6.** Expression pattern of the *empty-spiracles.* gene for the three *A. cerana.* embryo ages. (TIFF 114 kb)

Additional file 18: **Table S12.** Comparison of our *A. cerana.* Day1 embryonic transcriptome with the previously reported *A. mellifera.* Day1 embryonic transcriptome. (XLSX 10 kb)

Additional file 19: **Table S13.** GO terms enriched with the specific genes expressed in the first-day embryos of *A. cerana.* and *A. mellifera.*. (XLSX 25 kb)

Additional file 20: **Figure S7.** Comparison of the glycolysis/gluconeogenesis pathways enriched by the identified genes by the transcriptomic (A) and proteomic (B) methods. (TIFF 819 kb)

## Abbreviations

ANOVA: Analysis of variance
cDNA: complementary deoxyribonucleic acid
CRISPR/Cas9: Clustered regularly-interspaced short palindromic repeats/clustered regularly-interspaced short palindromic repeats associated protein 9
DEGs: Differentially expressed genes
FPKM: Expected number of fragments per kilobase of transcript sequence per millions base pairs sequenced
GO: Gene ontology
h: Hour
KEGG: Kyoto encyclopedia of genes and genomes
Mb: Million base
mRNA: message ribonucleic acid
MS: Mass spectrometry
NGS: Next-generation sequencing
ORF: Open reading frame
PCA: Principal component analysis
qRT-PCR: quantitative real time polymerase chain reaction
RNA-seq: RNA Sequencing
tRNA: transfer ribonucleic acid.
