# Supplementary figures and images for: Dynamic transcriptome landscape of Asian domestic honeybee (Apis cerana) embryonic development revealed by high-quality RNA sequencing
Source: BMC Dev Biol. 2018 Apr 13;18:11. doi: 10.1186/s12861-018-0169-1 (PMC5899340; doi:10.1186/s12861-018-0169-1)

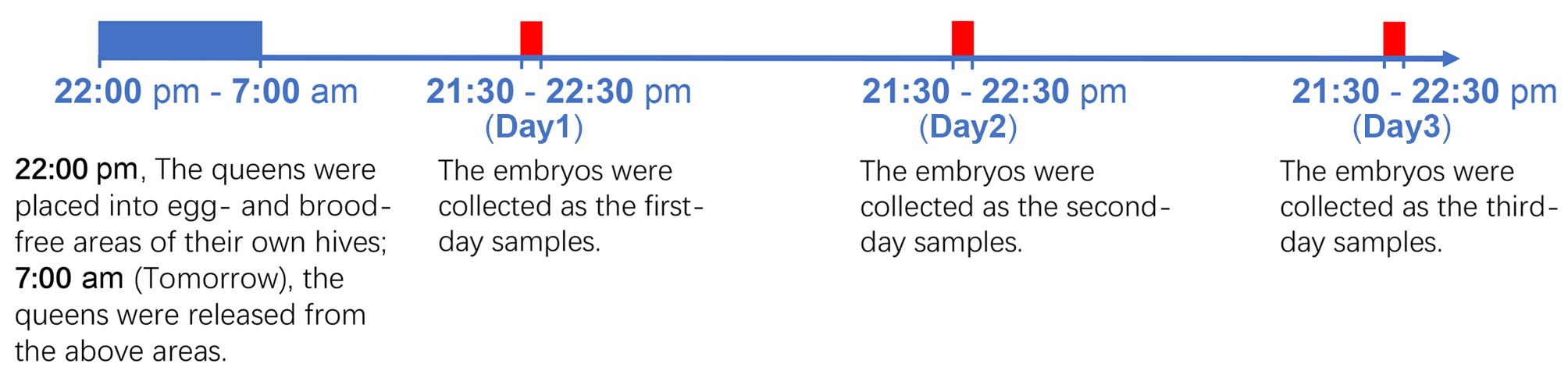

Supplement: Supplementary file 1 — Figure S1. Collection of the A. cerana. embryo samples. (TIFF 214 kb) [file 12861_2018_169_MOESM1_ESM.tif]

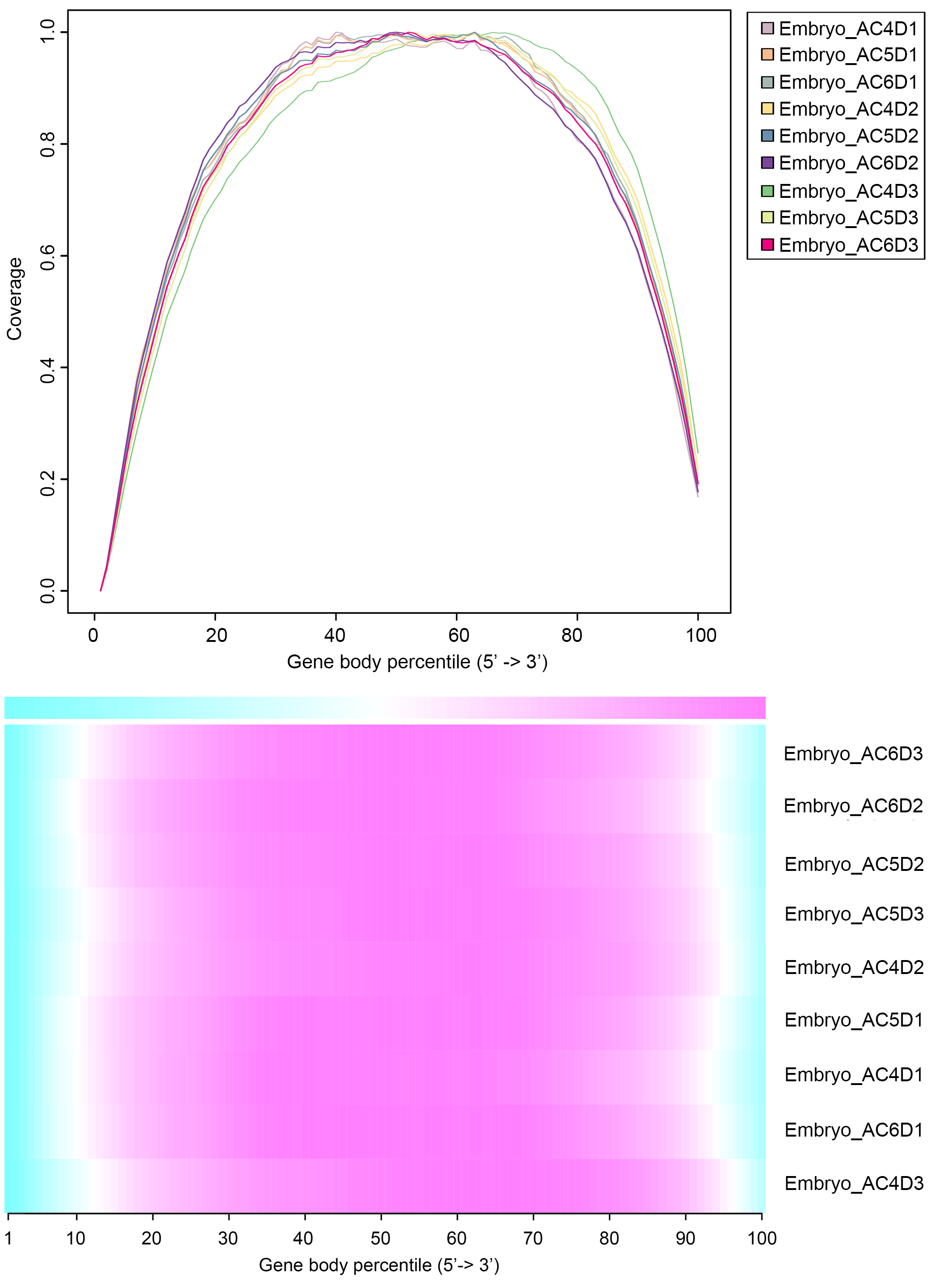

Supplement: Supplementary file 6 — Figure S2. Gene body coverage of all the embryo samples. These results show that the RNA-seq libraries and RNA-seq data of our embryo samples were good enough and were suitable for the subsequent bioinformatic analyses. (TIFF 965 kb) [file 12861_2018_169_MOESM6_ESM.tif]

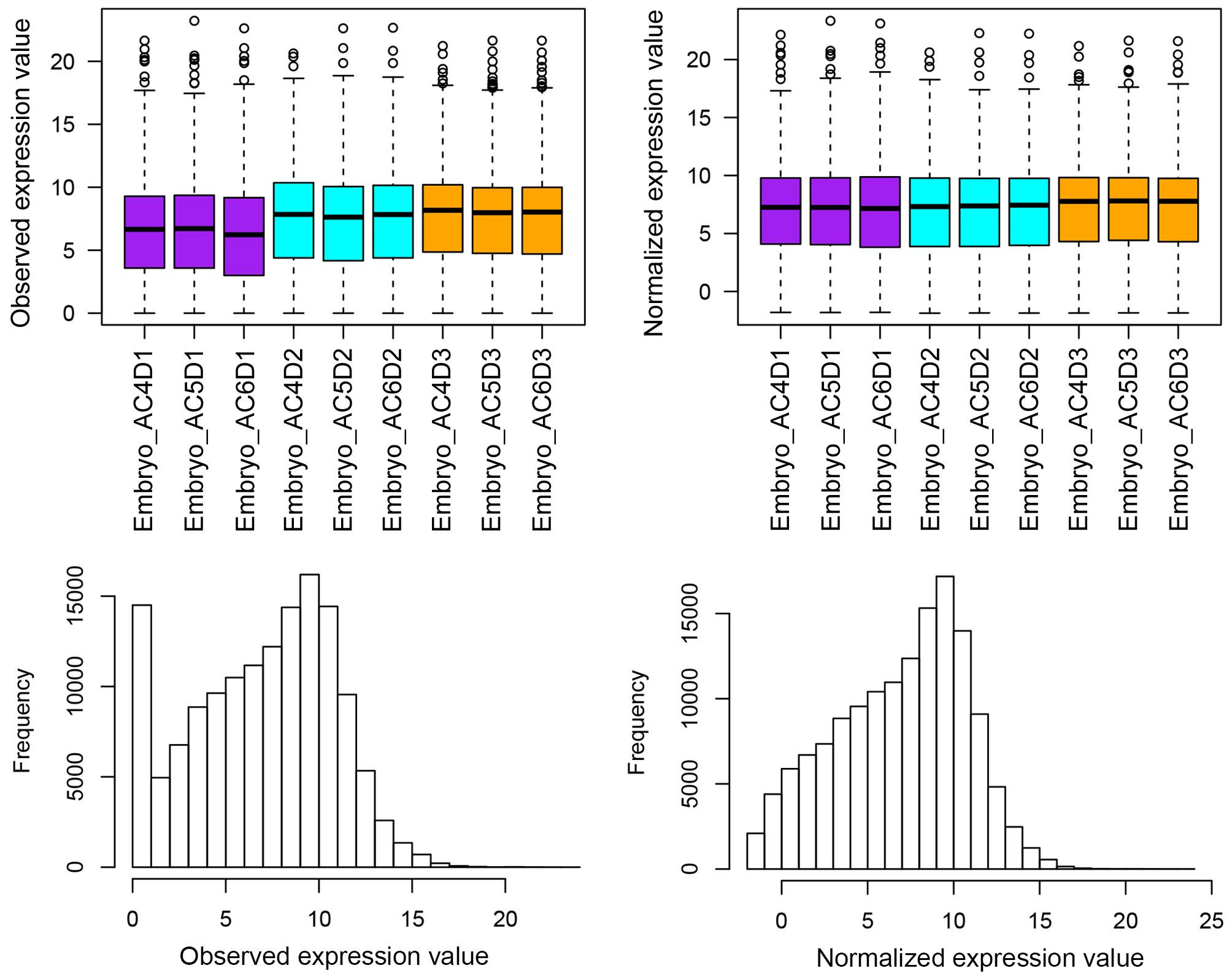

Supplement: Supplementary file 7 — Figure S3. Unnormalized and normalized transcript expression levels of our embryo samples and seven other tissues of A. cerana. downloaded from the NCBI database. (TIFF 410 kb) [file 12861_2018_169_MOESM7_ESM.tif]

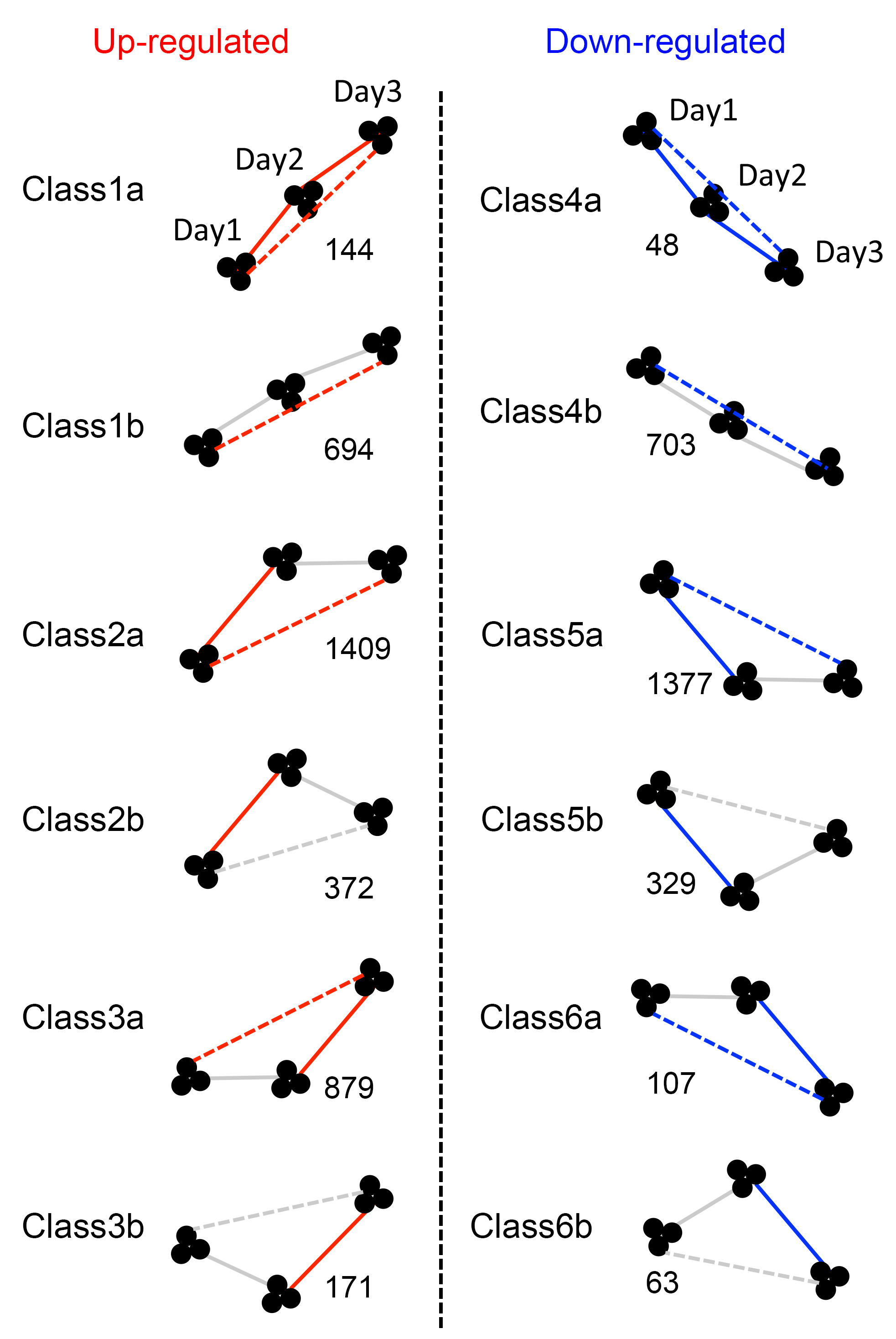

Supplement: Supplementary file 14 — Figure S4. Expression pattern of differentially expressed genes. These differentially expressed genes were categorized into six expression model classes. (TIFF 345 kb) [file 12861_2018_169_MOESM14_ESM.tif]

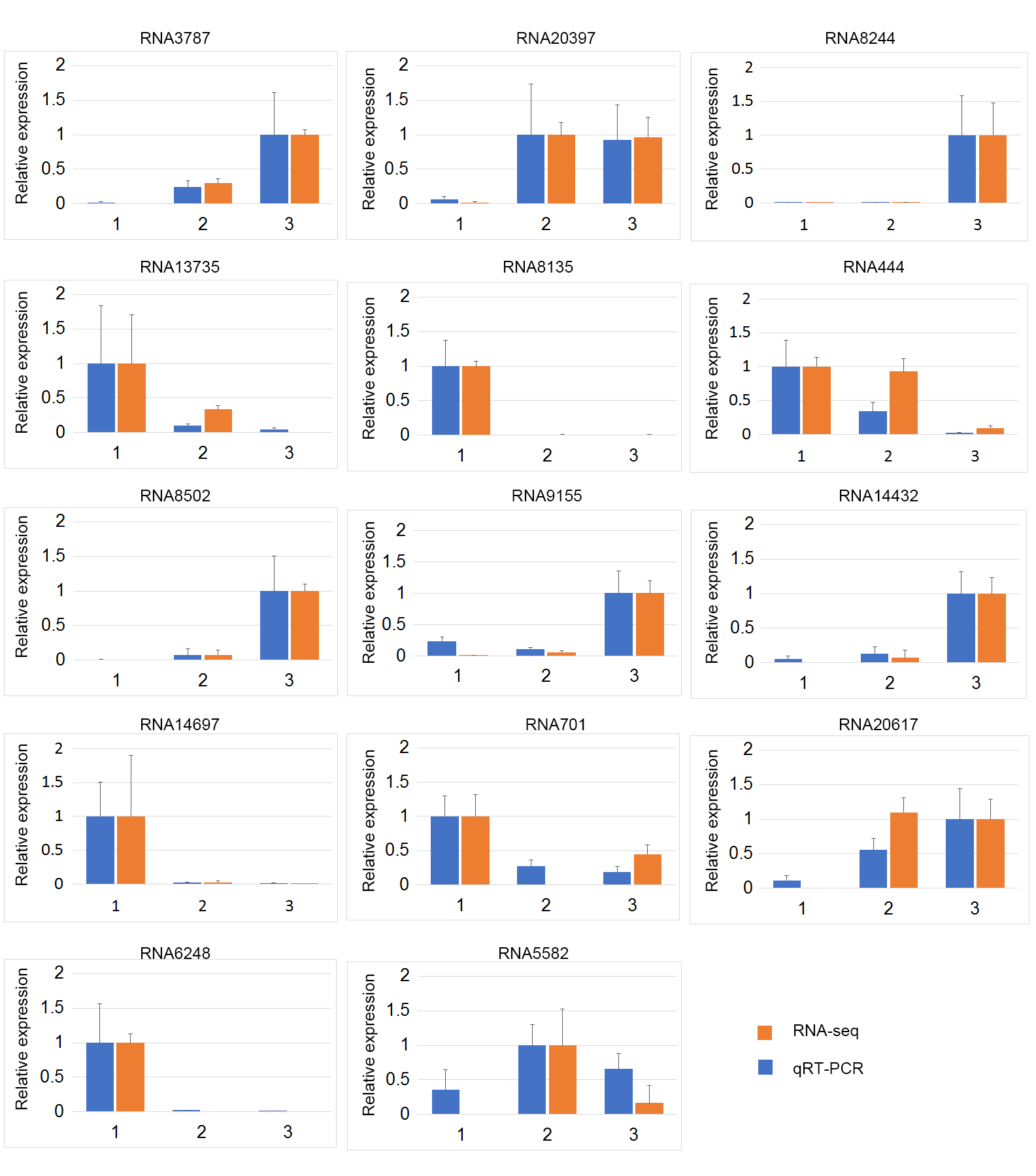

Supplement: Supplementary file 15 — Figure S5. Comparison between the RNA-seq and RT-qPCR results for 14 differential expressed genes identified by the R statistical package DEseq2. Most of the differential expressed genes were validated by ANOVA, and their expression patterns based on qRT-PCR were generally consistent with the ones based on RNA-seq. (TIFF 170 kb) [file 12861_2018_169_MOESM15_ESM.tif]

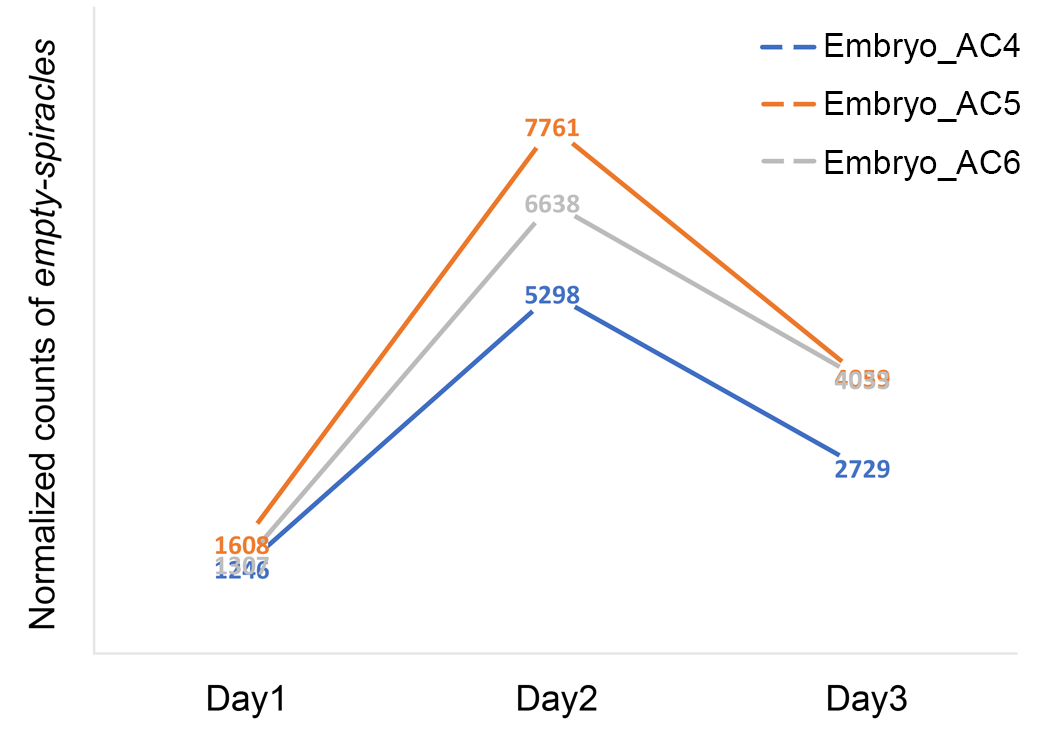

Supplement: Supplementary file 17 — Figure S6. Expression pattern of the empty-spiracles. gene for the three A. cerana. embryo ages. (TIFF 114 kb) [file 12861_2018_169_MOESM17_ESM.tif]

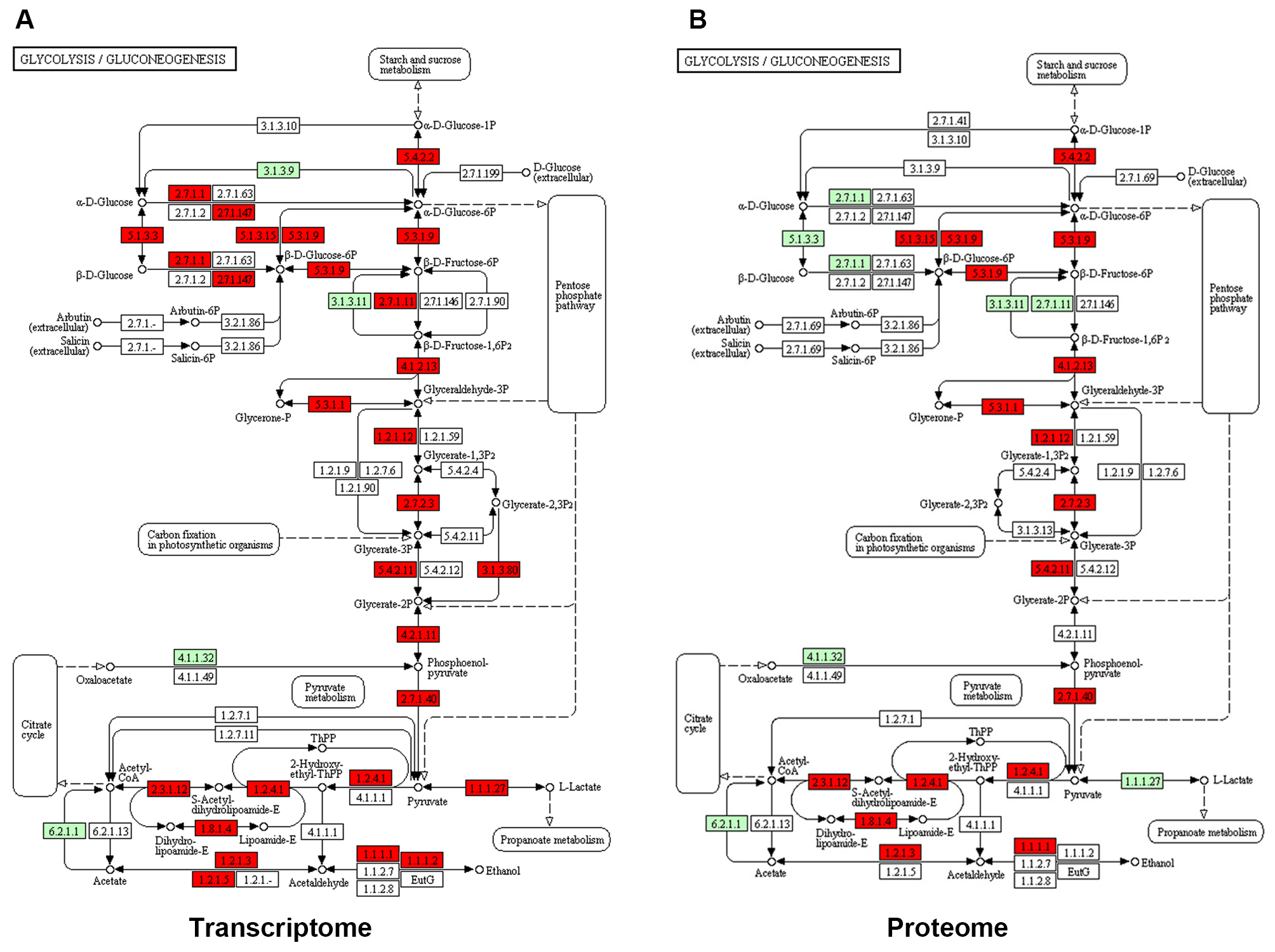

Supplement: Supplementary file 20 — Figure S7. Comparison of the glycolysis/gluconeogenesis pathways enriched by the identified genes by the transcriptomic (A) and proteomic (B) methods. (TIFF 819 kb) [file 12861_2018_169_MOESM20_ESM.tif]
