# Supplementary material for: Dynamic transcriptome landscape of Asian domestic honeybee (Apis cerana) embryonic development revealed by high-quality RNA sequencing
Source: BMC Dev Biol. 2018 Apr 13;18:11. doi: 10.1186/s12861-018-0169-1 (PMC5899340; doi:10.1186/s12861-018-0169-1)
Supplement: Supplementary file 2 — Table S1. Differentially expressed genes and their primers for qRT-PCR. (DOCX 19 kb) [file 12861_2018_169_MOESM2_ESM.docx]

**Additional file 2: Table S1. Differentially expressed genes and their primers for qRT-PCR**

| **No** | **Gene** | **Left primer** | **Right primer** |
| --- | --- | --- | --- |
| 1 | Rna9155 (Neurotrypsin) | CGAACCACCTGACCAAGAAT | CGAATCTTTTCCTTGGTGGA |
| 2 | Rna5582 (epidermal growth factor receptor isoform X2) | TGCCGTGTTTGATTGTGATT | TTCATAGACGCGCGTTACAC |
| 3 | Rna14432 (protein trachealess isoform X5) | TCCCTCCTGCTATTCCCTTT | CTCGAGGAGAGATGGACGAG |
| 4 | Rna14697 (serine/arginine repetitive matrix protein) | GAGCAACAACGTTGGCAATA | AGCACGTTCTTCTCGATGGT |
| 5 | Rna3787 (PREDICTED: probable chitinase 3) | CAATGAAAGGCCAGCTCAAT | TGCAGAGAGTCGGATCAATG |
| 6 | Rna8502 (alpha-glucosidase isozyme II) | CGACCTGGGGACTCTTGTAG | GGACGGTATAGGCGATTTGA |
| 7 | Rna20397 (PREDICTED: protein sister of odd and bowel) | ACCTGAGGAGGCACTCATTG | AGGACGTCTCTTGCAGCACT |
| 8 | Rna20617 (PREDICTED: branchpoint-bridging protein-like isoform X1) | AAACGATAACGACCGTGGAG | GCGTTCATTGGCTTCGTATC |
| 9 | Rna701 (PREDICTED: probable serine/threonine-protein kinase roco9) | TTTTGTTCATCTTCAAATTCATCAT | TTCAGAACCAAAAACTTCTGATATT |
| 10 | Rna8135 (Inactive serine/threonine-protein kinase TEX14) | GGGGATTCATCAGCACAAAT | TTAAATTCCGATCCTCAAAGC |
| 11 | Rna6248 (PREDICTED: glycosyltransferase-like protein LARGE1 isoform X1) | GCCAGTGTTAAAGCCTCGTC | AGCGCTAGGGTTGACAGAAA |
| 12 | Rna8244 (PREDICTED: uncharacterized protein LOC107995161 isoform X2) | GGTTCCTCGGTGTAGAGCAG | TTCGACATCGACAAGCAGAC |
| 13 | Rna444 (PREDICTED: G1/S-specific cyclin-E1) | ATCATTTCCTTGGGCTGATG | GTTGGGTGCCTTTGAAACAT |
| 14 | Rna13735 (uncharacterized protein LOC107998436 isoform X1) | CACGCGTCTCCTTACCAAAT | AGAACGACGGAAACAAGTGG |
| 15 | Actin 3 | GGCTCCCGAAGAACATCC | TGCGAAACACCGTCACCC |
