# Supplementary material for: Dynamic transcriptome landscape of Asian domestic honeybee (Apis cerana) embryonic development revealed by high-quality RNA sequencing
Source: BMC Dev Biol. 2018 Apr 13;18:11. doi: 10.1186/s12861-018-0169-1 (PMC5899340; doi:10.1186/s12861-018-0169-1)
Supplement: Supplementary file 3 — Table S2. RNA-seq statistics of collected embryo samples. (DOCX 14 kb) [file 12861_2018_169_MOESM3_ESM.docx]

**Additional file 3: Table S2. RNA-seq statistics of collected embryo samples.**

| **Sample** | **Collection_time** | **Raw_read** | **Clean_read** | **Data_size^1^** | **GC (%)** | **Q20 (%)** |
| --- | --- | --- | --- | --- | --- | --- |
| Embryo_AC4D1 | Day 1 | 65495004 | 57039476 | 8555921400 | 36.82 | 95.13 |
| Embryo_AC5D1 | Day 1 | 65494956 | 57611598 | 8641739700 | 36.49 | 94.96 |
| Embryo_AC6D1 | Day 1 | 63857602 | 56886490 | 8532973500 | 36.73 | 94.98 |
| Embryo_AC4D2 | Day 2 | 67132486 | 57675848 | 8651377200 | 37.18 | 95.32 |
| Embryo_AC5D2 | Day 2 | 65493708 | 57917142 | 8687571300 | 37.63 | 95.14 |
| Embryo_AC6D2 | Day 2 | 65494900 | 56886310 | 8532946500 | 37.91 | 95.25 |
| Embryo_AC4D3 | Day 3 | 68769648 | 57206742 | 8581011300 | 40.06 | 95.88 |
| Embryo_AC5D3 | Day 3 | 65494752 | 57512458 | 8626868700 | 40.76 | 95.68 |
| Embryo_AC6D3 | Day 3 | 63857504 | 56650514 | 8497577100 | 40.75 | 95.67 |
| ^1^Data_size: the size of clean read data | | | | | | |
