# Supplementary material for: Dynamic transcriptome landscape of Asian domestic honeybee (Apis cerana) embryonic development revealed by high-quality RNA sequencing
Source: BMC Dev Biol. 2018 Apr 13;18:11. doi: 10.1186/s12861-018-0169-1 (PMC5899340; doi:10.1186/s12861-018-0169-1)
Supplement: Supplementary file 5 — Table S4. Mapping statistics of RNA-seq data. (DOCX 14 kb) [file 12861_2018_169_MOESM5_ESM.docx]

**Additional file 5: Table S4. Mapping statistics of RNA-seq data.**

| **Sample** | **Collection time** | **Unmapped reads** | **Mapping rate (%)** | **Properly paired (%)** | **Singleton (%)** | **Splice reads (%)** |
| --- | --- | --- | --- | --- | --- | --- |
| Embryo_AC4D1 | Day 1 | 13904169 | 75.6 | 78.3 | 15.0 | 26.7 |
| Embryo_AC5D1 | Day 1 | 14961342 | 74.0 | 76.8 | 16.5 | 27.4 |
| Embryo_AC6D1 | Day 1 | 14886648 | 73.8 | 76.2 | 16.7 | 26.1 |
| Embryo_AC4D2 | Day 2 | 15015310 | 74.0 | 80.5 | 13.5 | 28.2 |
| Embryo_AC5D2 | Day 2 | 16186550 | 72.1 | 79.5 | 14.4 | 28.4 |
| Embryo_AC6D2 | Day 2 | 15138580 | 73.4 | 80.0 | 13.9 | 28.2 |
| Embryo_AC4D3 | Day 3 | 14222765 | 75.1 | 83.0 | 9.8 | 30.1 |
| Embryo_AC5D3 | Day 3 | 14766029 | 74.3 | 82.7 | 11.2 | 29.1 |
| Embryo_AC6D3 | Day 3 | 13851756 | 75.5 | 82.9 | 10.7 | 30.6 |
