# Supplementary material for: Dynamic transcriptome landscape of Asian domestic honeybee (Apis cerana) embryonic development revealed by high-quality RNA sequencing
Source: BMC Dev Biol. 2018 Apr 13;18:11. doi: 10.1186/s12861-018-0169-1 (PMC5899340; doi:10.1186/s12861-018-0169-1)
Supplement: Supplementary file 8 — Table S5. Summary of the embryonic transcriptomes (FPKM > 1) and comparison with the annotated A. cerana. reference genes. (DOCX 17 kb) [file 12861_2018_169_MOESM8_ESM.docx]

**Additional file 8: Table S5. Summary of the embryonic transcriptomes (FPKM > 1) and comparison with the annotated *A. cerana* reference genes.**

| **Code** | **Description** | **Day 1** | | | **Day 2** | | | **Day 3** | | |
| --- | --- | --- | --- | --- | --- | --- | --- | --- | --- | --- |
|  |  | **Embryo_AC4D1** | **Embryo_AC5D1** | **Embryo_AC6D1** | **Embryo_AC4D2** | **Embryo_AC5D2** | **Embryo_AC6D2** | **Embryo_AC4D3** | **Embryo_AC5D3** | **Embryo_AC6D3** |
| T | Total transcript | 13864 | 14312 | 13313 | 17727 | 17360 | 17300 | 18611 | 17857 | 17565 |
| L | Loci of transcript | 8771 | 9057 | 8475 | 10544 | 10730 | 10421 | 11625 | 11505 | 10983 |
| tpl | Transcripts per loci | 1.58 | 1.58 | 1.57 | 1.68 | 1.62 | 1.66 | 1.60 | 1.55 | 1.60 |
| = | Complete match of intron chain | 6574 | 6713 | 6325 | 8203 | 7766 | 8239 | 8162 | 7796 | 8196 |
| j | Potentially novel isoform (fragment): at least one splice junction is shared with a reference transcript | 3688 | 3780 | 3399 | 5082 | 4641 | 4777 | 4814 | 4388 | 4400 |
| u | Unknown, intergenic transcript | 1304 | 1335 | 1208 | 1732 | 1833 | 1699 | 2047 | 2264 | 2040 |
| i | A transfrag falling entirely within a reference intron | 613 | 687 | 579 | 714 | 999 | 627 | 1193 | 1114 | 821 |
| c | Contained | 589 | 654 | 738 | 693 | 776 | 708 | 938 | 938 | 788 |
| p | Possible polymerase run-on fragment (within 2Kbases of a reference transcript) | 346 | 360 | 309 | 426 | 463 | 425 | 488 | 465 | 494 |
| e | Single exon transfrag overlapping a reference exon and at least 10 bp of a reference intron, indicating a possible pre-mRNA fragment. | 321 | 374 | 322 | 346 | 356 | 321 | 383 | 381 | 331 |
| x | Exonic overlap with reference on the opposite strand | 240 | 246 | 236 | 293 | 302 | 272 | 292 | 245 | 249 |
| o | Generic exonic overlap with a reference transcript | 183 | 159 | 194 | 231 | 220 | 229 | 288 | 262 | 244 |
| s | An intron of the transfrag overlaps a reference intron on the opposite strand (likely due to read mapping errors) | 6 | 4 | 3 | 7 | 4 | 3 | 6 | 4 | 2 |
