# Supplementary material for: Dynamic transcriptome landscape of Asian domestic honeybee (Apis cerana) embryonic development revealed by high-quality RNA sequencing
Source: BMC Dev Biol. 2018 Apr 13;18:11. doi: 10.1186/s12861-018-0169-1 (PMC5899340; doi:10.1186/s12861-018-0169-1)
Supplement: Supplementary file 9 — Table S6. KEGG pathways enriched with novel genes identified in the embryonic development. (DOCX 14 kb) [file 12861_2018_169_MOESM9_ESM.docx]

**Additional file 9: Table S6. KEGG pathways enriched with novel genes identified in the embryonic development.**

| **Term** | **ID** | **Input number** | **Background number** | ***P*-Value** | **Corrected *P*-Value** | **Entrez Gene ID Input** |
| --- | --- | --- | --- | --- | --- | --- |
| Phototransduction - fly | dme04745 | 6 | 27 | 5.39E-05 | 0.0020 | 48632\|36003\|37368\|3355013\|40148\|41885 |
| Hippo signaling pathway - fly | dme04391 | 8 | 59 | 7.32E-05 | 0.0020 | 33245\|48632\|34179\|37368\|41273\|41783\| 42896\|41885 |
| Phagosome | dme04145 | 9 | 77 | 7.48E-05 | 0.0020 | 48632\|44307\|37368\|37238\|41166\|317846\| 34587\|40848\|41885 |
| ECM-receptor interaction | dme04512 | 4 | 11 | 0.0002 | 0.0040 | 33726\|33727\|38723\|31612 |
| Oxidative phosphorylation | dme00190 | 11 | 139 | 0.0003 | 0.0050 | 44307\|19893549\|46069\|19893556\|19893551\| 19893540\|317846\|19893535\|19893533\|34587\|43507 |
| Metabolic pathways | dme01100 | 34 | 924 | 0.0017 | 0.0196 | 41173\|36209\|34552\|36927\|46069\|32585\| 19893556\|19893551\|19893535\|19893533\| 192507\|43507\|37118\|44307\|33373\|31532\| 40469\|34587\|32285\|33642\|38723\|19893549\| 317846\|43620\|38746\|19893540\|34021\|39396\|31423\| 42402\|32334\|37884\|43512\|50392 |
| Endocytosis | dme04144 | 8 | 123 | 0.0062 | 0.0498 | 35900\|42665\|3355013\|41840\|42501\|39542\| 33025\|41551 |
